# Association Mapping of Germination Traits in *Arabidopsis thaliana* Under Light and Nutrient Treatments: Searching for G×E Effects

**DOI:** 10.1534/g3.114.012427

**Published:** 2014-06-05

**Authors:** Ginnie D. Morrison, C. Randal Linder

**Affiliations:** Department of Integrative Biology, University of Texas at Austin, Austin, Texas

**Keywords:** *A. thaliana*, germination, GWAS, environmental effects, natural genetic variation

## Abstract

In the natural world, genotype expression is influenced by an organism’s environment. Identifying and understanding the genes underlying phenotypes in different environments is important for making advances in fields ranging from evolution to medicine to agriculture. With the availability of genome-wide genetic-marker datasets, it is possible to look for genes that interact with the environment. Using the model organism, *Arabidopsis thaliana*, we looked for genes underlying phenotypes as well as genotype-by-environment interactions in four germination traits under two light and two nutrient conditions. We then performed genome-wide association tests to identify candidate genes underlying the observed phenotypes and genotype-by-environment interactions. Of the four germination traits examined, only two showed significant genotype-by-environment interactions. While genome-wide association analyses did not identify any markers or genes explicitly linked to genotype-by-environment interactions, we did identify a total of 55 markers and 71 genes associated with germination differences. Of the 71 genes, four—ZIGA4, PS1, TOR, and TT12—appear to be strong candidates for further study of germination variation under different environments.

When a seed germinates often determines the environmental conditions a plant will face, thus affecting its lifetime fitness. Many species use environmental cues to either initiate germination or remain dormant, and the reliability of these cues often provides a fitness advantage (Donohue *et al.* 2005a). Reliable cues are especially important for annual plants (Rees and Long 1992; Donohue *et al.* 2005a), which have only one opportunity to reproduce after a single growing season and, therefore, less time for environmental conditions to change after germination.

Environmental heterogeneity may select for different seed germination characteristics within a single species. Selection for different characteristics can result in at least two outcomes: adaptive plasticity (Via and Conner 1995) or local adaptation due to genotype-by-environment interactions (G×E). In the case of adaptive plasticity, selection operates such that a single genotype produces different, adaptive phenotypes depending on the environment. For example, some species accommodate environmental heterogeneity via bet-hedging, *i.e.*, different seeds with the same genotype, from the same maternal plant, have different germination requirements (Simons and Johnston 2006; Simons 2009). Local adaptation can result from G×E if a genotype expresses different phenotypes due to environmental conditions (Lynch and Walsh 1998), but selection favors the phenotype produced in one environment but not the other phenotype in another environment (Ungerer *et al.* 2003). Populations with two or more genotypes may become locally adapted to multiple habitats by these means or by simply having genotypes with different, unvarying phenotypes that are selectively favored in different environments.

Because annual plants are usually unable to change location after germination, selection for local adaptation of germination via phenotypic plasticity or G×E may be common, as it would produce germination phenotypes appropriate in the home environment.

Light and nutrients affect germination timing and total germination in many weedy ephemeral species (Hilhorst and Karssen 1988; Adler *et al.* 1993; Weinig 2010). Light can strongly affect germination timing and cuing in some plant species by allowing a seed to sense whether it is buried or overtopped by neighbors (Rees and Brown 1991; Schmitt and Wulff 1993; Baskin and Baskin 2006). Although less well-studied, nutrient availability in the environment may also influence germination because germinating under higher nutrient concentrations may confer a fitness advantage (Hilhorst and Karssen 1988). Because nutrient availability often varies seasonally, it could also be a reliable cue for germination timing (Chapin 1980).

*Arabidopsis thaliana* exhibits significant variation in germination timing and total amount of germination (Donohue *et al.* 2005a,b; Schmuths *et al.* 2006; Boyd *et al.* 2007; Huang *et al.* 2010). Quantitative trait loci (QTL) studies have revealed chromosomal locations important for germination responses under different environmental conditions (Vanderschaar *et al.* 1997; Alonso-Blanco *et al.* 2003; Schmuths *et al.* 2006; Laserna *et al.* 2008; Meng *et al.* 2008; Bentsink *et al.* 2010; Huang *et al.* 2010; Silady *et al.* 2011). However, these studies have been limited to identifying broad chromosomal regions containing tens to hundreds of genes and to regions where genetic variation existed between the mapping parents. Knockout and mutant studies have shown that the uptake and assimilation of nitrate in *Arabidopsis thaliana* seeds affect germination success and are influenced by at least four genes (Alboresi *et al.* 2005; Chopin *et al.* 2007; Finch-Savage *et al.* 2007).

Genome-wide association studies (GWAS) can partially overcome the problems associated with QTL studies by using the natural variation present in a large number of geographically and genetically distinct individuals from many populations. Further, GWAS use much denser marker sets than traditional QTL studies and capture more recombination events, therefore identifying smaller chromosomal ranges for loci influencing a trait under particular conditions. To date, few GWAS using *A. thaliana* have looked at germination traits (Atwell *et al.* 2010; Derose-Wilson and Gaut 2011).

Here, we present the results of a GWAS on four germination traits of 100 natural *A. thaliana* accessions and a publicly available set of 213,624 SNPs (Nordborg 250K dataset). We assessed these traits under fully factorial light and nutrient combinations. Our goal was to identify genetic regions associated with germination timing and total proportion of seeds germinated under different environmental conditions and to assess whether traits were subject to G×E effects.

## Materials and Methods

### Accession selection

**Figure 1:**
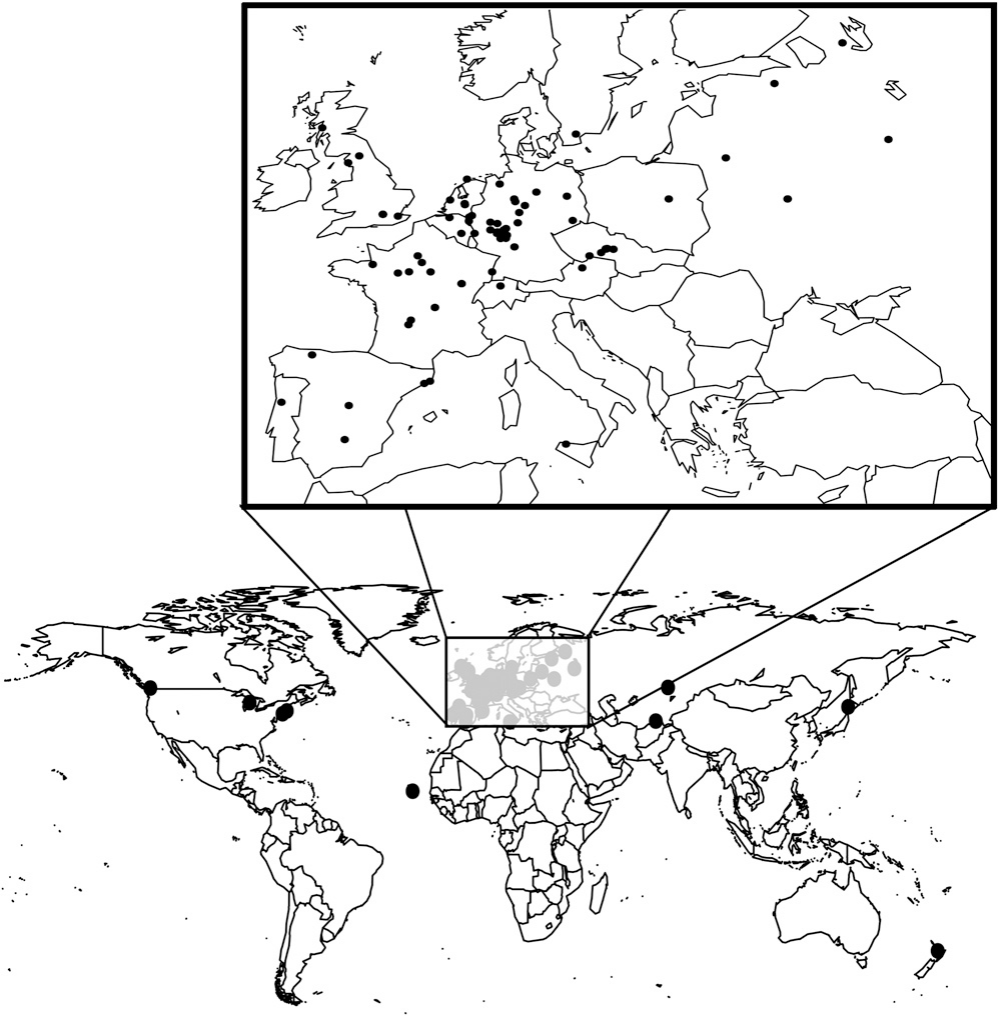
Locations of the 100 natural accessions used in this study.

We used 100 accessions from the Arabidopsis Biological Resource Center (ABRC, http://abrc.osu.edu/) (Figure 1 and Supporting Information, Table S1). All accessions were part of an earlier version of the *A. thaliana* RegMap panel (version 3.05, http://archive.gramene.org/db/diversity/diversity_view) (Horton *et al.* 2012). We chose accessions from an initial screen of 167 accessions. From this screen, we chose 100 accessions that flowered without a period of vernalization. Accessions in the initial screen were determined not to need vernalization if they bolted within 1 month of the earliest bolting plants. This minimized possible genetic differences due to seasonal germination variation (Donohue *et al.* 2005c; Weinig *et al.* 2002). At the same time, variation that affects both flowering time and germination may not have been sampled. Accessions originated from latitudes ranging from 37.79°S to 61.36°N and longitudes ranging from 123°W to 141.35°E (Table S1 and Figure 1).

### Seed generation

To minimize environmental maternal effects and produce seed for the experiment, we grew the accessions under common garden conditions. We stratified seeds in 500 µL of ddH_2_O at 4° for 7 d and then planted them in MetroMix 200 potting soil. Each accession had eight replicates. Plants were germinated in two growth chambers (Percival Scientific, Inc.) with identical settings (15 hr light, 22° and 9 hr dark, 18°). We randomized pots within flats and then rotated flats weekly in the growth chambers. Each growth chamber had an equal number of representatives of each accession. We removed pots with no germination or seedlings that died. Before bolting began, the number of pots was small enough to place the surviving plants in a single chamber. All plants flowered and produced seed in one chamber, minimizing chamber effects on the seeds.

Beginning 2 wk after planting, we fertilized the plants every other week with a half-strength solution of Peter’s Professional 20-20-20 (∼125 ppm). We cupped and sleeved plants as soon as they bolted (Aracon System) and stopped watering when the plants senesced. After the plants dried, we harvested seeds and stored them in coin envelopes in the dark at room temperature for at least 30 d.

### Germination trial

We implemented a fully factorial design of two nutrient treatments (High and Low) and two light treatments (Full-Light and Dark). The Low treatment used one-sixteenth strength (∼16 ppm) Peter’s Professional 20-20-20 in ddH_2_O and the High treatment used one-eighth strength (∼33 ppm). Nutrient levels were selected based on previous trials using *A. thaliana* that showed these nutrient differences had germination effects (G. Morrison, unpublished data). Complete darkness (Dark) and full-spectrum light (Full-Light) were chosen for the light treatments because germination can occur at different rates and in different proportions under these conditions (Adler *et al.* 1993; Vanderschaar *et al.* 1997; Meng *et al.* 2008). We did not have a foliar-shade treatment due to space constraints.

Due to time and space constraints, the experiment was temporally blocked. Each block included two replicates of each factorial combination for each accession, and the blocks were conducted 2 wk apart. Overall, we had 1600 experimental units (100 accessions × 4 treatment combinations × 2 blocks × 2 replicates/block).

All seeds were surface-sterilized for 8 min in a 3.5% (v/v) sodium hypochloride solution with Triton-X as a surfactant and then rinsed three times with filter-sterilized ddH_2_O. The experimental unit was a 50-mm Petri dish (BD Falcon). Each dish held 25 seeds in a 5×5 array on doubled P7 filter paper (Fischer). We added 750 µL of sterile Low or High nutrient solution to each prepared plate. All plates were placed on trays in two black acrylic boxes and stratified at 4° for 3 d to 4 d. After stratification, trays were moved to a Percival growth cabinet (15 hr light, 22° and 9 hr dark, 18°); Full-Light plates were removed from the boxes under safe green light (Roscolux Moss Green Filter by Rosco) and placed in full light. Dark plates remained in the boxes in the growth chamber. Photographs of individual plates were taken under a safe green light every 12 hr for 10 d and then every 24 hr for the next 4 d using a Canon EOS digital camera with a macro lens. Plates were removed prior to the end of the experiment if all seeds had germinated or if mold was observed [16 of 1600 experimental units (1%) had mold; no treatment had a significantly higher amount of infection than any other; χ^2^ = 4.45, df = 3, *P* = 0.22].

### Phenotyping

Photographs were scored for germination. We considered a seed germinated when we observed its radicle protruding from the seed coat. Germination traits analyzed were final proportion germinated (FPG) at the end of the 2-wk period, time of first germinant (TFG), and two germination dynamics parameters of a germination curve fitted to each plate. The curve had the form of:

$$g\left(t;\;k,\;tmax,\;gmax\right)=\;gmax*e^{\left(\frac{k*\left(t-tmax\right)}{k*\left(t-tmax\right)+1}\right)}$$

where *g* is the proportion of seeds germinated at time *t*. The model estimates the maximum number of seeds germinated (*gmax*), the maximum rate of germination (*k*), and the time of the maximum germination rate (*tmax*). Values for the three parameters were estimated using the nls() function in R. For analysis, we were only interested in *k* and *tmax* because FPG was a direct measure of the final proportion of seeds germinated because the 2-wk germination period was sufficient to observe full germination of nondormant seeds for nearly all replicates. We only included maximum germination rate and time of maximum germination rate estimates with *P* < 0.05 in our linear models. The nls() function calculates *P* values from the estimated model’s profile likelihoods. Plates removed at any point due to mold were excluded from all analyses.

### Linear models for germination timing and total proportions

We explored the effects of genotype, environment, and G×E using generalized linear mixed models (GLMMs) for FPG and TFG and linear models for the two parameters describing germination dynamics. The distributions for FPG and TFG were non-normal (Shapiro-Wilkes test: FPG, W = 0.52, *P* < 0.001; TFG, W = 0.71, *P* < 0.001). Neither an arcsine square root transformation for the FPG data nor a box-cox transformation [bcpower in the car() package (Fox and Wiesburg 2011)] of TFG resulted in more normal residuals (Shapiro-Wilkes test: FPG, W = 0.62, *P* < 0.001; TFG, W = 0.85, *P* < 0.001). Therefore, for all analyses, we used untransformed data. The GLMM used a binomial distribution for FPG (as it is a proportion) and a Poisson distribution for TFG. In general, the full models were:

Y=L+N+A+L*N+L*A+N*A+B+Error,

where *L* (light quality: Full-Light or Dark) and *N* (nutrient level: High or Low) were fixed effects and *A* (accession) and *B* (block) were random effects. We did not analyze the three-way interaction because the three-way models did not converge. To determine the simplest, most explanatory model, we sequentially reduced each model by removing the least significant term and then comparing the −2 log likelihood score of the reduced model to the previous model. All models were run using lmer() in the R package lme4 (Bates and Maechler 2009).

The distributions of significant maximum germination rates and times of maximum germination rate, and their residuals, were also all non-normal (Shapiro-Wilkes test: time of maximum germination rate, W = 0.85, *P* < 0.001; maximum germination rate W = 0.97, *P* < 0.001). We found no transformations appropriate for the data, so we ran linear mixed-models on the untransformed data. However, because the mixed-model assumption of normal residuals was violated, interpretation of the results needs to be performed with care. The initial models used and the methods to determine the best reduced models were the same as for the TFG and FPGs.

### Heritability

We calculated the broad-sense heritabilities (H^2^) of the traits for each factorial combination using the SAS proc mixed procedure (SAS Institute Inc. 2008). The model *Y* = accession + block + error, with accession and block as a random effects, was used to obtain the variation due to genotype (accession) and total (accession + block + error) variance. The model fit with the accession term was compared with that without accession to test for a significant, genotypic effect. Although the data were not normal, we considered H^2^ significant if the difference between the −2 log likelihoods for the two models was greater than 3.84 (χ^2^ value significant at α = 0.05). Most differences were much greater than 3.84.

### Geographic location and germination

Genetic variation in *A. thaliana* shows structure based on geographic location (Beck *et al.* 2008; Zhao *et al.* 2007). This variation is often correlated with longitude or latitude and, hence, with the kinship matrix used in association mapping (see below). Therefore, important phenotypic variation associated with genetic variation on latitudinal or longitudinal clines might be masked by the kinship matrix (Zhao *et al.* 2007; Aranzana *et al.* 2005). To see if there was a correlation between the measured phenotypes and latitude or longitude, we ran two linear models testing each geographic feature separately. Generally, these models were:

Y=L+N+H+V+L*N*H*V+Error

where *Y* is the phenotypic value, *L* and *N* are as described before, and *H* and *V* represent the latitude and longitude at an accession’s reported collection site. For simplicity, L*N*H*V represents all possible two-way, three-way, and four-way interactions.

### Candidate genes

Prior to association mapping, we created a list of 132 *a priori* candidate genes known to be functionally important during germination or identified in previous studies of natural variation for germination traits (Table S2 and File S1). Based on gene function, we expected that four genes might be subject to G×E under different nutrient conditions and 36 genes might be subject to G×E under different light conditions. We considered the remaining 92 genes to be “general” germination genes, with no explicit hypotheses about which factors they would be most responsive to.

### Association mapping of individual SNPs

We used only FPG and time of maximum germination rate (TMAX) for association mapping because TFG and the maximum germination rate showed no significant G×E effects in the linear models. To assess whether the multi-trait mixed model method (MTMM) (Korte *et al.* 2012) or the simpler EMMA method (Kang *et al.* 2008) was most appropriate for our data, we calculated the phenotypic correlations between environments for phenotypes with a significant G×E interaction. We used Kendall’s tau because the data were not bivariate normal and contained ties. FPG values measured within each light environment were highly correlated (Dark/Low *vs.* Dark/High: Kendall’s tau = 0.71, *P* < 0.001; Full-Light/Low *vs.* Full-Light/High: Kendall’s tau = 0.76, *P* < 0.001), as were values within nutrient treatments (Dark/Low *vs.* Full-Light/Low: Kendall’s tau = 0.46, *P* < 0.001; Dark/High *vs.* Full-Light/High: Kendall’s tau =0.48, *P* < 0.001). TMAX values within light environments and within nutrient treatments also were correlated (Dark/Low *vs.* Dark/High: Kendall’s tau = 0.57, *P* < 0.001; Full-Light/Low *vs.* Full-Light/High: Kendall’s tau = 0.36, *P* < 0.001; Dark/Low *vs.* Full-Light/Low: Kendall’s tau = 0.35, *P* < 0.001; Dark/High *vs.* Full-Light/High: Kendall’s tau =0.47, *P* < 0.001). These results indicated MTMM was the better method to disentangle G×E effects (Korte *et al.* 2012).

Using the SNP data from version 3.05 of the Nordborg dataset, we implemented the MTMM described by Korte *et al.* (2012) to perform the association analyses. The MTMM method uses a K-matrix, created with EMMA (Kang *et al.* 2008), to control for population structure. For the 2×2 factorial set-up used in this study, MTMM first runs a model for each of the four factorial environments (individual models), which are simply GWA analyses for each treatment combination (Full-Light/High, Full-Light/Low, Dark/High, and Dark/Low). MTMM also runs five complex models: (1) a model testing for significant effects of both genotype and environment against a null model (full); (2) a model testing for significance of genotype alone against a null model (genotype only); (3) a model testing for G×E effects for light and nutrients together (G×E); (4) a model testing G×E for one factor (*e.g.*, light, G×L); and (5) a model testing G×E for the other factor (*e.g.*, nutrient, G×N) (Korte *et al.* 2012).

We removed all minor alleles (SNPs with frequencies ≤0.1) before the MTMM analyses because minor alleles are prone to spuriously low *P* values (Atwell *et al.* 2010). We controlled experiment-wise type I error by correcting the raw *P* values with a Benjamini-Hochberg false discovery rate (BH-FDR) correction (Benjamini and Hochberg 1995) in R using the p.adjust() function (The R Project for Statistical Computing).

To test for significant candidate genes, we used the MTMM model on two sets of SNPs: (1) only the SNPs present within 1000 bp upstream of a candidate gene’s start site and 500 bp downstream of a candidate gene’s stop site (a highly constrained view of the gene, ignoring linkage) and (2) all SNPs within 10 kb upstream and downstream of a gene (the average size of haplotype blocks in *A. thaliana*) (Kim *et al.* 2007)

To perform a genome-wide analysis of germination, we then used the MTMM model to look for associations between the full set of SNPs, minus minor alleles, and either FPG or TMAX.

### Association mapping of reaction norms

To find SNPs associated with phenotypic reactions to the light and nutrient treatments, we performed four association-mapping analyses in EMMA. The four analyses were: light response under low nutrients; light response under high nutrients; nutrient response under full-light; and nutrient response under darkness. Responses were calculated as:

(predicted phenotypic value under one light or nutrient condition) − (predicted phenotypic value under the other condition).

As above, analyses used genotypes with no minor alleles, and *P* values were corrected using a BH-FDR.

### Genes linked to significant SNPs

Using PLINK (version 1.07) (Purcell *et al.* 2007), we calculated the pair-wise linkage disequilibrium (LD) between SNPs within 20-kb windows (Kim *et al.* 2007). Only pairs with an r^2^ ≥ 0.2 were considered linked. We accepted a gene as linked to a significant SNP if that gene encompassed at least one SNP found to be in LD with a significant SNP. For genes linked to significant SNPs, we used available TAIR10 gene descriptions and gene ontologies (GOs) to identify gene function and to assess whether linked genes had any known effect on germination. We also used Amigo’s GO Term Enrichment Tool (version 1.8) (Carbon *et al.* 2009) to identify any GO terms for which our genes were enriched.

### Candidate gene enrichment

We performed tests of enrichment for each of the MTMM models tested as a way to evaluate the false-positive rate. Enrichment was calculated as in Atwell *et al.* (2010). Within each model, we permuted the *P* values 10,000 times and calculated the proportion of the top 50, 100, 250, 500, 1000, and 5000 SNPs linked to a candidate gene. SNPs were considered linked as per above. *P* values for the observed proportion of SNPs linked to candidates were calculated as the number of permuted proportions with a value less than or equal to the observed proportion plus one divided by the number of permutations plus one.

## Results

### Germination results

**Figure 2:**
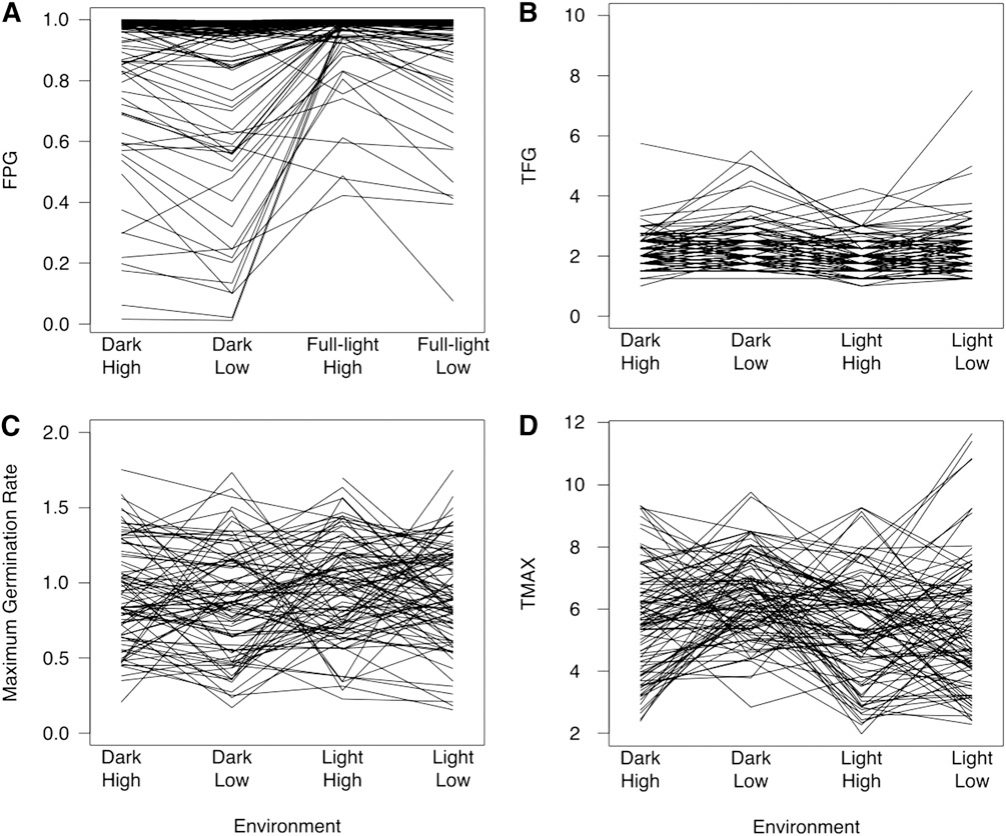
Plots of each accession’s (line’s) mean trait value under the four factorial environments for each germination characteristic. (A) FPG = final proportion germinated. (B) TFG = time of first germinant in days. (C) Maximum germination rate (proportion of seeds germinated per day). **(D)** TMAX = time of maximum germination rate in days.

The average final proportion germination (FPG) for each treatment ranged from 80.7% ± 0.27 to 94.5% ± 0.11; however, not all lines reached a high FPG and both rank and scale changes were observed (Figure 2A). Significantly fewer plates in Full-Light had <50% total germination than plates in the darkness (45 of 793 in Full-Light and 120 of 791 in Dark; χ ^2^ = 30.8; df = 1; *P* < 0.0001). Accessions having plates with germination proportions <80% likely had dormant seeds because the same accessions had germination proportions ≥81% when independent sets of seeds were treated with gibberillic acid (GA) to force germination (data not shown). It could be that these accessions preferred a warm/wet stratification rather than a cold/wet one. The TFG did not vary greatly between the four factorial combinations (1.02–1.18 d) (Figure 2B).

For the germination dynamics measures, we were able to fit curves for 1543 of 1584 experimental units. A total of 948 plates had significant estimated maximum germination rates, and 1525 plates units had significant estimated TMAXs. The maximum germination rate ranged from 0.06 to 1.75, measured as the proportion of seeds germinated per day (Figure 2C). Numbers greater than 1 indicate that all seeds germinated in less than 1 d. TMAX ranged from 1.53 to 29.13 d (Figure 2D).

Broad-sense heritabilities ranged widely, depending on the germination trait, but nearly all were significant. All FPG and TFG heritabilities were significant. Within factorial combinations, broad-sense heritabilities for FPG (0.44–0.55) (Table 1) were higher than those for TFG (0.16–0.26). Heritabilities for maximum germination rate were also significant in all cases (range, 0.25–0.37) and fell between the values for FPG and TFG. Significant heritabilities of TMAX fell within a similar range (0.27–0.32) as those for the maximum germination rate, and only the heritability under the Full-Light/Low treatment (H^2^ = 0.10) was not significant.

**Table 1:** Broad-sense heritability for each factorial combination and phenotype

Phenotype	Treatment	>H^2^
FPG	Dark/Low	**0.55**
	Dark/High	**0.47**
	Full-Light/High	**0.47**
	Full-Light/Low	**0.44**
TFG	Dark/Low	**0.26**
	Dark/High	**0.16**
	Full-Light/High	**0.21**
	Full-Light/Low	**0.22**
MGR	Dark/Low	**0.37**
	Dark/High	**0.33**
	Full-Light/High	**0.32**
	Full-Light/Low	**0.25**
TMAX	Dark/Low	**0.31**
	Dark/High	**0.27**
	Full-Light/High	**0.32**
	Full-Light/Low	0.10

Bold heritabilities are significant at *P* ≤ 0.05. H^2^, broad-sense heritability; MGR, Maximum germination rate.

### Phenotype and G×E effects

Two G×E effects (light×accession and nutrient×accession) were significant for FPG, as were the light and nutrient main effects (Table 2). Full-Light and High nutrients resulted in higher values for FPG. For TFG, the nutrient, light, and accession effects were all significant, but no G×E effects were significant. Full-Light and High nutrients both caused TFG to occur earlier. For the germination dynamics measures, there was a significant light×accession effect for TMAX as well as significant light and nutrient effects. Full-Light and High caused the maximum germination rate to occur sooner than the Dark or Low treatments. The maximum germination rate was influenced by accession and light and nutrient conditions. Full-Light and High nutrients increased the maximum germination rate. Because there were only significant G×E effects for FPG and TMAX, we continued analyses only for those two phenotypes.

**Table 2:** GLMM models and their AIC and log likelihood scores

Model*^a^*^,^*^b^*	AIC	Log Likelihood	Significant Fixed Terms
**TFG**			
Y∼L+N+L*N+A+L*A+N*A+B	693.8	−334.9	
Y∼L+N+L*N+A+L*A+N*A	724	−351	
Y∼L+N+L*N+A+L*A+B	687.8	−334.9	
Y∼L+N+L*N+A+B	681.8	−334.9	
Y∼L+N+L*N+B	727.9	−359	
**Y∼L+N+A+B**	**680.3**	**−335.1**	**Earlier germination in Full-Light and High**
			
**MGR**			
Y∼L+N+L*N+A+L*A+N*A+B	892.27	−433.13	
Y∼L+N+A+L*A+N*A+B	890.58	−433.29	
Y∼L+N+A+L*A+B	885.86	−433.93	
**Y∼L+N+A+B**	**884.60**	**−436.30**	**Lower max rate under Dark and under Low**
			
**TMAX**			
Y∼L+N+A+L*N+L*A+N*A+B	7840	−3908.00	
Y∼L+N+L*N+A+L*A+N*A	**7842**	**−3908.00**	
			
**FPG**			
Y∼L+N+L*N+A+L*A+N*A+B	**6222**	**−3103**	**Higher proportions in Full-Light/High**
Y∼L+N+L*N+A+L*A+B	6508	−3247	
Y∼L+N+L*N+N*A+A+B	7980	−3983	

a Where Y = phenotype measured, L = light treatment, N = nutrient treatment, A = Arabidopsis accession, B = block.

b Models in bold are those determined to be the best based on AIC and likelihood criteria. MGR, Maximum germination rate.

### Geographic location and FPG

There was no association between FPG and either latitude (F_1,1579_ = 0.99, *P* = 0.32) or longitude (F_1,1579_ = 0.58, *P* = 0.45). The same held true for TMAX (latitude: F_1,1519_ = 1.01, *P* = 0.32; longitude: F_1,1519_ = 0.18, *P* = 0.67).

### Significant candidate gene SNPs

All candidate genes had SNPs within 10 kb upstream and downstream of their start and stop sites, respectively. A total of 4030 different SNPs were within the range examined (3–87 SNPs/gene; average, 31.7). When the analysis was restricted to SNPs located within genes, three of the original 132 candidates were not represented. A total of 762 SNPs were found within 129 genes (1–33 SNPs/gene; average, 5.91).

For FPG at an FDR level of 0.05, we found three significant SNPs associated with four candidate genes in a total of four models (full, genotype only, G×L, and Full-Light/Low). One SNP, which was significant in the full and genotype-only models (adjusted *P* = 0.03 and 0.01, respectively), was in *TT12* (At3g59030), a transparent testa gene. TT12 affects testa pigmentation and permeability and might affect light penetration (due to testa pigmentation) and nutrient availability (due to testa permeability). The same SNP was also within 10 kb of candidate gene *PIL6* (At3g59060), a phytochrome-interacting gene. A second SNP, which was significant in the full and G×L model (adjusted *P* = 0.03 and 0.02, respectively), was linked with candidate gene *RAS1* (At1g09950). RAS1 has no described function but has been confirmed to be involved in salt-tolerant and ABA-insensitive germination (Ren *et al.* 2010). Finally, the candidate gene *NRT2.7* had a significant SNP in the Full-Light/Low model (adjusted *P* = 0.01). NRT2.7 is involved in nitrogen transport in seeds.

When only intragenic SNPs were considered, only the SNP identified in *TT12* remained significant. It was significant in the full and genotype-only models, as well as the Full-Light/High model (corrected *P* = 0.006, 0.002, and 0.032, respectively). While the SNP was linked with *PIL6*, it was not within *PIL6*.

For TMAX, we found two significant SNPs associated with three candidate genes in a total of two models (Full-Light/Low and Full-Light/High). In the Full-Light/High model, the same SNP found in *TT12* and linked to *PIL6* for FPG was significant (corrected *P* = 0.025 and corrected *P* < 0.0001, respectively). In the Full-Light/Low model, the same SNP found in *NRT2.7* for FPG was significant in the Full-Light/Low model (corrected *P* = 0.025). When only intragenic SNPs were considered, two candidate-gene SNPs were significant. Again, the SNP in *TT12* was significant, this time in the genotype-only and the Full-Light/High models (corrected *P* = 0.013 and corrected *P* < 0.0001, respectively). The second SNP was within the candidate gene *FRS2* (At2g32250), which responds to red/far-red light, for the Full-Light/High model. The function of FRS2 is unknown. It is also unclear why this SNP was not significant when the longer range was considered.

### Significant genome-wide SNPs

#### FPG:

**Figure 3:**
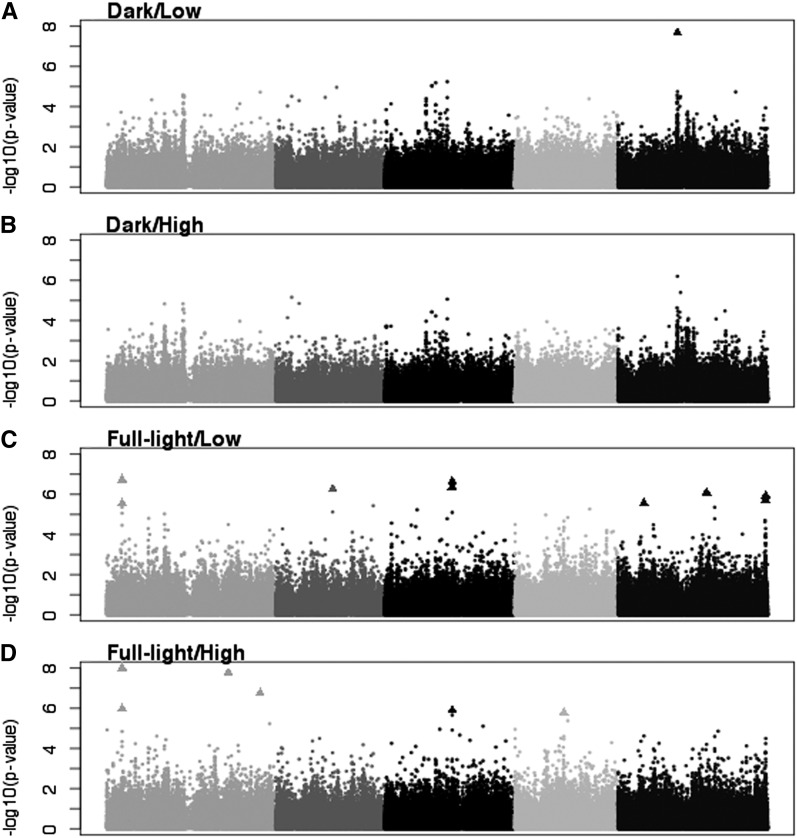
Manhattan plots of uncorrected *P* values for the four individual models for FPG. Triangles represent SNPs significant at the 0.05 level after Benjamini-Hochberg correction. (A) Dark/Low. (B) Dark/High. (C) Full-Light/Low. (D) Full-Light/High.

**Figure 4:**
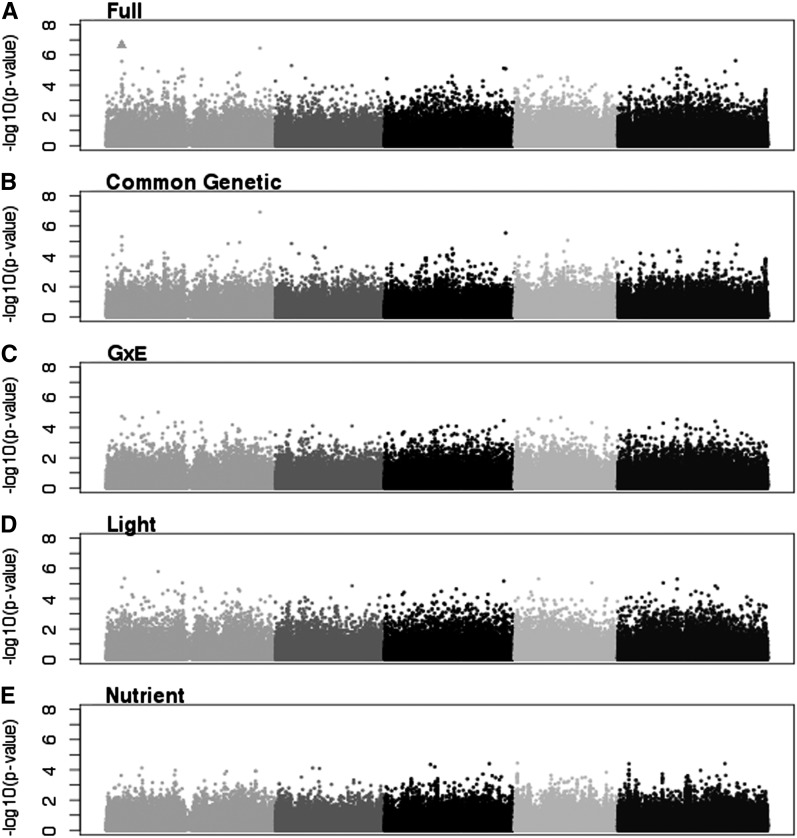
Manhattan plots of uncorrected *P* values for FPG in the (A) Full, (B) genotype only, (C) G×E, (D) GxL, and (E) G×N models. Triangles represent SNPs significant at the 0.05 level after Benjamini-Hochberg correction.

The MTMM analysis identified 14 significant SNPs associated with FGP (Table 3 and raw scores in File S2). Some of these SNPs were likely linked because they were within 10 kb of each other (Table 3). In some cases, putatively linked significant SNPs had intervening nonsignificant SNPs (Figure 3 and Figure 4).

**Table 3:** The 25 SNPs significant for FPG in at least one of the nine MTMM models

	Models								
SNP	Dark/Low	Dark/ High	Full-Light/Low	Full-Light/High	Full	Genotype Only	G×E	G×L	G×N
**Chr1:2757164**	3.11E−03	0.024	1.82E−07*^b^*	1.05E−06*^b^*	2.64E−06	1.85E−05	5.62E−03	5.33E−03	0.0514
Chr1:2759471	0.091	0.319	4.35E−06*^a^*	2.19E−04	8.74E−05	1.61E−03	3.27E−03	3.70E−03	0.0372
**Chr1:2763016**	0.261	0.369	8.66E−06*^a^*	4.54E−05	9.41E−05	1.50E−03	3.77E−03	9.09E−04	0.434
**Chr1:2765047**	0.029	0.049	1.16E−09*^b^*	9.63E−09*^b^*	2.14E−7*^b^*	4.88E−06	1.50E−03	3.22E−04	0.521
**Chr1:2770350**	0.012	0.012	2.86E−06*^b^*	1.42E−05	1.34E−04	3.89E−05	0.164	0.0628	0.876
**Chr1:10419017**	5.09E−04	1.83E−03	9.30E−06*^a^*	7.41E−05	2.80E−04	5.83E−05	0.243	0.126	0.379
**Chr1:21916027**	7.42E−03	0.014	3.18E−05*^a^*	1.65E−08*^b^*	6.60E−05	1.44E−05	0.201	0.0862	0.764
**Chr1:27668561**	1.87E−05	3.60E−04	1.31E−09*^b^*	1.79E−07*^b^*	3.49E−07*^b^*	1.15E−7*^b^*	0.0907	0.109	0.0932
**Chr2:10297188**	0.064	0.17	5.42E−07*^b^*	1.65E−04	1.21E−04	1.02E−03	6.94E−03	3.51E−03	0.122
Chr2:10297285	0.076	0.19	7.50E−06*^a^*	1.64E−03	8.22E−04	2.94E−03	0.0198	0.0154	0.0892
**Chr2:17620611**	0.188	0.228	3.63E−06*^a^*	1.80E−04	2.33E−04	2.52E−03	6.03E−03	1.49E−03	0.46
Chr3:5837328	0.025	0.096	5.82E−06*^a^*	1.38E−03	2.21E−04	1.16E−03	0.0116	0.023	0.0276
Chr3:12162344	0.177	0.248	7.91E−06*^a^*	1.21E−05	2.81E−04	6.42E−04	0.0258	7.37E−03	0.494
**Chr3:12162371**	0.082	0.116	4.25E−07*^b^*	1.23E−06*^b^*	2.49E−05	2.07E−04	5.91E−03	1.38E−03	0.595
**Chr3:12163116**	0.01	0.024	2.23E−07*^b^*	2.15E−06*^a^*	6.59E−05	3.09E−05	0.0976	0.0355	0.464
Chr4:5556326	0.02	0.078	1.06E−05*^a^*	1.48E−04	3.30E−04	3.24E−04	0.0578	0.0471	0.119
**Chr4:8843014**	0.014	0.053	2.12E−05	1.64E−06*^b^*	1.05E−04	4.65E−05	0.107	0.0624	0.226
Chr4:9533814	6.03E−04	4.32E−03	1.42E−05	4.17E−06*^a^*	4.76E−05	8.61E−06	0.234	0.171	0.24
Chr4:13491707	0.049	0.138	5.36E−06*^a^*	1.59E−04	2.19E−04	6.61E−04	0.0193	0.0135	0.101
**Chr5:4690632**	0.015	0.034	2.76E−06*^b^*	2.38E−05	1.86E−04	3.38E−04	0.0305	0.0112	0.299
**Chr5:10723903**	2.07E−08*^b^*	6.21E−07	8.24E−04	0.027	9.93E−05	3.84E−05	0.122	0.117	0.261
Chr5:15976193	0.032	0.108	7.93E−07*^b^*	6.21E−05	4.62E−05	8.58E−04	2.99E−03	1.99E−03	0.0702
Chr5:17435459	0.029	0.068	4.36E−06*^a^*	1.02E−04	4.18E−04	6.30E−04	0.0399	0.0159	0.281
								
**Chr5:26628368**	0.034	0.053	1.89E−06*^b^*	5.66E−05	1.94E−04	2.57E−04	0.0401	0.0116	0.668
Chr5:26647798	3.12E−03	6.63E−03	1.15E−06*^b^*	3.15E−05	3.18E−04	1.83E−04	0.0949	0.0316	0.585

Raw *P* values are shown for each SNP in each model. SNP names in bold are also significant for TMAX SNPs significant at the 0.1 level

a 0.05 < *P* ≤ 0.1 after a B-H FDR correction.

b *P* ≤ 0.05 after a B-H FDR correction.

Twelve of the 14 significant SNPs were identified only in the individual models, and 10 were significant in only one individual model (Figure 3, Figure 4, and Table 3). One SNP was identified in four models (full, genotype only, Full-Light/Low, and Full-Light/High) and the other was significant in three of the four (full, Full-Light/Low, and Full-Light/High). These two SNPs are the most likely to be associated with environmental effects because of their significance in the full model, which tests both genotype and environment effects. No significant SNPs were detected in the models explicitly testing for G×E overall, G×L, or G×N.

**Figure 5:**
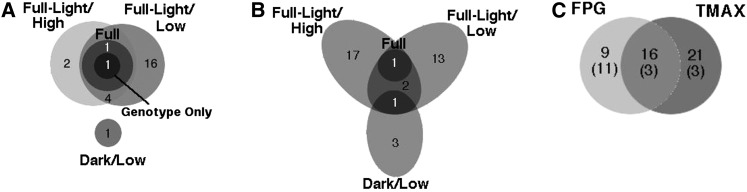
Venn diagrams representing significant SNPs (at FDR ≤0.1) shared among germination treatments (A, B) and characteristics (C). (A) Between models within FPG. (B) Between models within TMAX. (C) Between the FPG and TMAX phenotypes (numbers in parentheses are significant at the FDR ≤0.05 level).

Because GWAS is often treated as a hypothesis-generating tool, we also examined SNPs significant at an FDR of 0.1. Eleven new SNPs were significant in the Full-Light/Low individual model, and a twelfth SNP, previously significant at the 0.05 level, was significant in an additional model (Full-Light/High) (Figure 5A).

#### TMAX:

**Figure 6:**
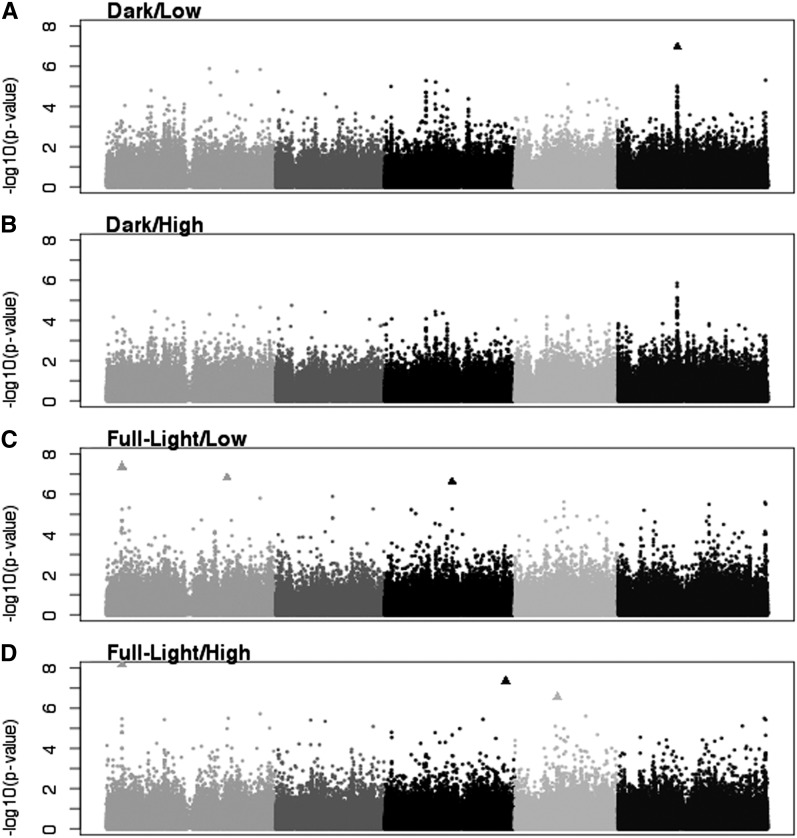
Manhattan plots of uncorrected *P* values for the four environment models for TMAX. Triangles represent SNPs significant at the 0.05 level after Benjamini-Hochberg correction. (A) Dark/Low. (B) Dark/High. (C) Full-Light/Low. (D) Full-Light/High.

**Figure 7:**
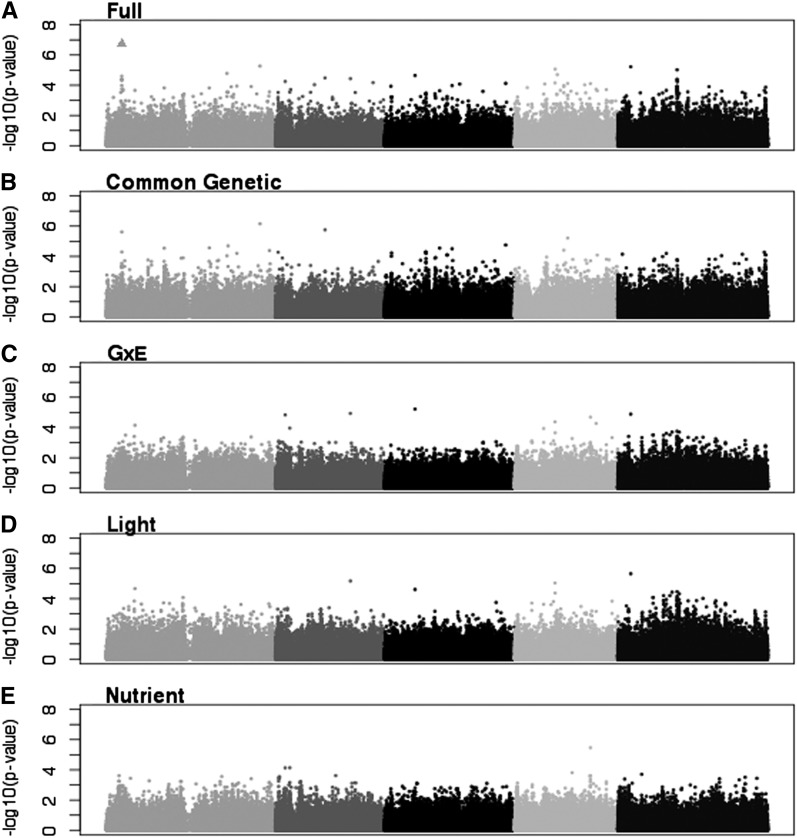
Manhattan plots of uncorrected *P* values for the four environment models for TMAX. Triangles represent SNPs significant at the 0.05 level after Benjamini-Hochberg correction. (A) Full. (B) Genotype only. (C) GxE. (D) GxL. (E) GxN.

At an FDR-corrected *P* value level of 0.05, there were six significant SNPs (Table 4 and raw scores File S3). They were significant in the full, Dark/Low, Full-Light/Low, and Full-Light/High models, and no SNPs were significant in any G×E models (Figure 6 and Figure 7). The Dark/Low model had one significant SNP that was not significant in any other model. The Full-Light/Low, Full-Light/High, and full models all shared one significant SNP, and the Full-Light/Low and Full-Light/High models each identified two different SNPs (Figure 6).

**Table 4:** The 37 SNPs significant for TMAX rate in at least one of the nine MTMM models

SNP	Dark/Low	Dark/ High	Full-Light/Low	Full-Light/High	Full	Genotype Only	G×E	G×L	G×N
**Chr1:2757164**	0.010	0.052	4.66E−05	3.35E−06*^a^*	2.51E−05	2.86E−04	0.004	0.011	0.069
**Chr1:2763016**	0.191	0.112	8.86E−05	7.26E−06*^a^*	4.43E−05	4.40E−04	0.005	0.002	0.607
**Chr1:2765047**	0.021	0.010	4.17E−08*^b^*	6.62E−09*^b^*	1.76E−07*^b^*	2.38E−06	0.002	0.001	0.443
**Chr1:2770350**	0.009	0.003	5.56E−06*^a^*	1.03E−04	1.76E−04	5.04E−05	0.171	0.274	0.161
Chr1:4058155	0.013	0.009	4.68E−06*^a^*	0.013	0.001	0.003	0.024	0.395	0.012
**Chr1:10419017**	3.63E−05	0.001	1.17E−04	3.63E−06*^a^*	1.50E−04	2.84E−05	0.249	0.344	0.206
Chr1:18526664	1.28E−06*^a^*	4.89E−05	0.002	2.38E−04	1.50E−04	2.70E−05	0.262	0.247	0.202
Chr1:21713582	0.025	0.017	1.53E−07*^b^*	1.03E−05*^a^*	1.63E−05	9.06E−05	0.008	0.011	0.133
**Chr1:21916027**	2.13E−04	0.001	3.60E−04	3.10E−06*^a^*	2.85E−04	2.00E−05	0.680	0.415	0.817
Chr1:23494937	1.77E−06*^a^*	5.49E−05	0.002	7.55E−04	1.13E−04	6.22E−05	0.088	0.247	0.047
**Chr1:27668561**	1.43E−06*^a^*	2.19E−05	1.55E−06*^a^*	1.86E−06*^a^*	5.32E−06	6.94E−07	0.267	0.962	0.108
Chr1:29368367	0.009	0.005	0.002	9.67E−06*^a^*	2.74E−04	4.01E−05	0.337	0.278	0.267
Chr2:6351897	0.017	0.032	1.40E−04	3.84E−06*^a^*	2.93E−04	3.99E−04	0.042	0.012	0.779
Chr2:8960447	2.37E−05	3.81E−05	7.38E−05	4.44E−06*^a^*	3.24E−05	1.73E−06	0.724	0.943	0.431
**Chr2:10297188**	0.028	0.019	1.27E−06*^a^*	7.48E−04	6.29E−04	0.001	0.030	0.054	0.104
**Chr2:17620611**	0.149	0.102	5.33E−06*^a^*	7.99E−06*^a^*	6.74E−05	5.09E−04	0.007	0.002	0.980
Chr3:4786505	0.084	0.024	5.79E−06*^a^*	2.05E−04	8.96E−04	7.93E−04	0.072	0.024	0.859
Chr3:5586649	0.016	0.011	9.14E−06*^a^*	3.90E−04	0.002	0.002	0.097	0.046	0.552
**Chr3:12162371**	0.017	0.013	5.28E−06*^a^*	2.27E−04	7.42E−04	5.80E−04	0.079	0.071	0.249
**Chr3:12163116**	0.003	0.002	2.36E−07*^b^*	2.14E−05	1.05E−04	3.11E−05	0.157	0.150	0.263
Chr3:13530762	0.364	0.211	0.004	1.02E−05*^a^*	8.30E−05	0.001	0.004	0.003	0.063
Chr3:17718905	0.025	0.020	0.001	3.54E−06*^a^*	2.54E−04	2.34E−04	0.059	0.028	0.258
Chr3:21818882	0.001	0.007	0.002	4.30E−08*^b^*	7.48E−05	1.75E−05	0.190	0.088	0.414
Chr4:7287800	0.596	0.379	1.45E−05	7.79E−06*^a^*	8.51E−06	0.002	2.21E−04	4.20E−05	0.523
Chr4:7657583	0.066	0.187	7.61E−05	2.77E−07*^b^*	1.97E−05	4.04E−04	0.002	5.92E−04	0.956
Chr4:8841131	0.076	0.016	2.38E−06*^a^*	3.91E−05	1.23E−04	3.84E−04	0.018	0.005	0.890
**Chr4:8843014**	0.006	0.005	1.24E−05	1.01E−05*^a^*	1.05E−04	3.85E−05	0.128	0.098	0.326
Chr4:8843150	0.198	0.026	5.17E−06*^a^*	1.92E−04	3.15E−04	3.51E−04	0.051	0.018	0.720
Chr4:12776709	0.009	0.048	0.001	2.45E−06*^a^*	3.22E−04	1.75E−04	0.100	0.072	0.316
**Chr5:4690632**	0.011	0.002	6.20E−06*^a^*	0.001	0.003	0.001	0.303	0.207	0.453
**Chr5:10723903**	1.07E−07*^b^*	7.86E−06	0.006	0.008	5.83E−05	1.90E−04	0.016	0.022	0.045
Chr5:16425024	0.008	0.001	3.13E−06*^a^*	0.001	0.001	3.54E−04	0.161	0.220	0.186
Chr5:22442725	0.001	0.005	7.81E−05	7.61E−06*^a^*	2.94E−04	7.22E−05	0.209	0.197	0.283
Chr5:26393336	0.002	0.004	9.82E−05	3.14E−06*^a^*	2.21E−04	5.27E−05	0.208	0.088	0.771
Chr5:26499148	0.056	0.047	2.52E−06*^a^*	0.002	0.001	0.003	0.018	0.058	0.056
**Chr5:26627873**	0.005	0.008	9.43E−05	3.71E−06*^a^*	3.05E−04	7.91E−05	0.200	0.083	0.767
Chr5:26628368	0.051	0.012	3.05E−06*^a^*	2.20E−05	1.36E−04	7.86E−05	0.086	0.033	0.725

Raw *P* values are shown for each SNP in each model. SNPs in bold are also significant F-SNPs at the 0.1 level.

a 0.05 < *P* ≤ 0.1 after a B-H FDR correction.

b *P* ≤ 0.05.

As with FPG, we also examined SNPs that were significant at an FDR of 0.1. We identified an additional 31 significant SNPs at this level (Table 4). The additional SNPs were distributed among the Dark/Low (3), Full-Light/Low (14), and Full-Light/High (17) models. The three models shared one significant SNP, and Full-Light/Low and Full-Light/High models shared one SNP (Figure 5B). Otherwise, each of the models had unique significant SNPs. One previously identified SNP was significant at the 0.1 level in the Full-Light/High model.

At the 0.05 level, three SNPs were significant for both FPG and TMAX, and an additional 13 were significant for both traits at the 0.1 level (Figure 5C).

### Gene enrichment for candidate-linked SNPs

Candidate gene enrichment was not detected for FPG or TMAX. Some models showed enrichment >1, but these values were not significant (Table S6 and Table S7). There was no clear pattern between the amount of enrichment and the number of top SNPs tested or model tested. Five candidate genes (*ABA1*, *PHYA*, *NCED6*, *SDP1*, and *TT1*) (Table S2) were not in LD with any SNP tested.

### Genes linked to genome-wide SNPs

#### FPG:

Twenty-six genes were linked, as per PLINK, to 15 of the 25 SNPs significant at an FDR level ≤0.1 (Table S3); 10 SNPs were not linked to a gene. Each linked SNP was linked to one to five genes (Table S3), and none was an *a priori* candidate gene. Seven genes had a significant SNP located within an exon, 3′UTR, or intron (Table 5). Ten of the 26 genes (linked to 12 of the 15 SNPs) were expressed in the seed or embryo (Lamesch *et al.* 2012), but none has a clear or known relationship with germination. However, the two genes linked to the SNP significant in the full model, *ZIGA4* (At1g08680) and *PS1* (At1g08700), are of interest because their general functions could be germination-related and they were expressed in the seed. ZIGA4 is involved in the regulation of GTPase activity, and PS1 is involved in signal transduction. The set of 26 genes linked to significant SNPs was not enriched for any particular molecular functions or biological processes.

**Table 5:** Genes with significant SNPs located within their transcribed regions

	Within Transcribed Gene			
Gene	Exon		Intron	3′UTR
AT1G08660	Chr1:2757164, Chr1:2759471			
AT1G08680			Chr1:2765047, Chr1:2763016	
AT1G08700	Chr1:2770350			
AT1G29750	Chr1:10419017			
AT1G50030			Chr1:18526664	
AT1G61890	Chr1:22870338			
AT1G63350	Chr1:23494937			
AT2G20815	Chr2:8960447			
AT2G24210	Chr2:10297188, Chr2:10297285			
AT2G24230	Chr2:10303780, Chr2:10303977			
AT3G14350			Chr3:4786505	
AT3G16440	Chr3:5586649			
AT3G23640			Chr3:8507820	
AT3G48000			Chr3:17718905	
AT4G08691	Chr4:5556326			
AT4G13180	Chr4:7657583			
AT4G15450	Chr4:8841131			
AT4G16940			Chr4:9533814	
AT4G18250	Chr4:10089582			
AT4G26800			Chr4:13491707	
AT5G07340	Chr5:2319344			
AT5G28690				Chr5:10723903
AT5G39890				Chr5:15976193
AT5G55350	Chr5:22442725			
AT5G65980		Chr5:26393336		
AT5G66740		Chr5:26647798		

#### TMAX:

Forty-nine genes were linked to 24 of the 37 SNPs significant at the 0.1 level (Table S4); 13 SNPs were not linked to a gene. Each SNP was linked to one to 10 genes, and one was linked with the *a priori* candidate, *TT12* (Full-Light/High model). Three SNPs were within an exon, intron, or 3′UTR of three different genes (Table 5). Thirty-four of the 49 linked genes were expressed in the seed or embryo and were linked to 20 of the 24 SNPs linked to at least one gene. *TOR* (At1g50030), linked to a SNP significant in the Dark/Low treatment, is involved in embryo development and could be a potential gene of interest. When compared with the GO of all TAIR genes, the genes linked to significant SNPs were enriched for the biological processes “transmembrane receptor protein tyrosine kinase signaling pathway” (*P* = 0.04; 4 genes) and “enzyme-linked receptor protein signaling pathway” (*P* = 0.04; 4 genes) and the molecular function “transferase activity” (*P* = 0.02; 14 genes, including TOR).

### Association mapping of reaction norms

#### FPG:

Three SNPs were significant at the 0.05 level for the nutrients-under-full-light reaction norm (Table 6). One was located within an exon of At2g24210, *Terpene Synthase 10*, an embryo-expressed gene. At the 0.1 level, nine different SNPs, distributed among chromosomes 1, 2, 3, and 5, were significant in the reaction norms tested (Table 6 and raw scores File S4).

**Table 6:** SNPs significant in the eight reaction norm models run

SNP	Phenotype	Model	*P*
Chr1:22870338	FPG	Nutrient/full	1.83e−06*^a^*
Chr1:27668561	FPG	Nutrient/full	4.00e−08*^b^*
Chr2:10297188	FPG	Nutrient/full	8.09e−07*^b^*
Chr3:5837328	FPG	Nutrient/full	2.91e−06*^a^*
Chr5:6399525	FPG	Nutrient/full	4.53e−07*^b^*
Chr5:15976193	FPG	Nutrient/full	2.19e−06*^a^*
Chr1:23494937	FPG	Nutrient/dark	4.88e−07*^a^*
Chr1:13726129	FPG	Light/low	2.18e−06*^a^*
Chr2:2177432	FPG	Light/low	5.12E−07*^a^*
Chr3:8507820	FPG	Light/low	1.79e−06*^a^*
Chr3:8511432	FPG	Light/low	1.79e−06*^a^*
Chr5:2319344	FPG	Light/low	2.75e−06*^a^*
Chr3:8507820	FPG	Light/high	7.25e−07*^a^*
Chr3:8511432	FPG	Light/high	7.25e−07*^a^*
Chr4:10089582	TMAX	Light/high	4.25e−07*^a^*

*P* values are uncorrected.

a 0.05 < *P* ≤ 0.1 after a B-H FDR correction.

b *P* ≤ 0.05 after a B-H FDR correction.

#### TMAX:

Only one SNP at the 0.1 FDR level was significant in any of the four TMAX reaction norms tested. This SNP was identified in the light-under-high-nutrients model (Table 6) and was located in an exon of At4G18250, a protein with phosphorylation abilities. Enrichment for GO terms was not tested for reaction norms genes due to the small number of linked genes (raw scores in File S5).

## Discussion

Determining the genetic causes of variation in germination across heterogeneous environments is important for our understanding of plant evolution and could have applications to agriculture. Considerable evidence shows that selection has often acted on seeds to use the information in light and, to a lesser extent, nutrients as germination cues, particularly in annuals (Schmitt *et al.* 1992; Weinig 2010; Adler *et al.* 1993; Hilhorst and Karssen 1988). Therefore, we expected measures of germination dynamics and final germination proportion to show G×E effects under different light and nutrient combinations. Germinating at high proportions in appropriate environments could result in higher fitness on average, and different environments, such as those with high competition or with shorter growing seasons, could select for different optimal germination dynamics after receiving a particular cue.

Only time of maximum germination rate and final germination proportion showed any evidence for G×E effects. Mixed models indicated significant interactions between genotype and light and nutrient conditions affecting both traits, and interaction plots of these two traits showed both changes in rank and response. However, no significant SNPs were identified in the models testing G×E. These results are, perhaps, not surprising given the results in a similar study by Korte *et al.* (2012). Using the same mixed model approach, they found only one significant SNP in one G×E model, even though their study included approximately four-times as many accessions (∼400 *vs.* 100). Korte *et al.* (2012) suggested that their inability to find G×E SNPs could have resulted from the complexity of the model. While G×E may be common, it can also be difficult to confirm due to small effect sizes and power issues (Des Marais *et al.* 2014; Smith and Kruglyak 2008; Korte *et al.* 2012). Within individual treatments, we were able to detect significant SNPs. This could be due to higher power and a larger response variance between accessions.

We might have also eliminated natural variation for G×E by examining only lines without a vernalization requirement. Twenty-six of the 132 candidate genes have a known role in flowering time or vernalization and some might be implicated in G×E effects in an experiment that included flowering time variants. Finally, it would be interesting to repeat this study with a foliar-shade treatment included because there may also be G×E effects associated with seed responses in this and our other light environments. Perhaps variation in responses between lines under foliar shade would be greater, thus increasing the power of the analysis.

### Candidate genes and enrichment

We expected our candidate genes would be linked to a large number of significant SNPs because of their associations with germination under other conditions, but this was largely not the case. We also observed no enrichment for SNPs linked to candidate genes. One reason some of our candidate genes might not have been detected by our study is that they might not vary in nature due to selective constraints. This is particularly likely for candidate genes whose functions were established using knockouts or laboratory-created mutants that could not survive in nature. Finally, the candidates we selected from prior association-mapping studies may not have influenced the germination traits we measured under our treatments (see below).

### Genes linked to SNPs

A total of 71 unique genes were linked to significant SNPs in the FPG, TMAX, and reaction norm analyses. Of these 71 unique genes, we identified four that were of particular interest based on their described roles in germination or relevant environment sensing. Two genes, *ZIGA4* and *PS1*, were identified in both the FPG and TMAX analyses and were significant at the 0.05 level in the full model for both phenotypes. While it is unknown if they play a role in germination, both are expressed in the seed, making them potential candidates. The other two genes (*TOR* and *TT12*) were identified only in the time of maximum germination analysis: *TOR* was identified in the Dark/Low individual model and *TT12*, an *a priori* candidate, was identified in the Full-Light/High model.

When we analyzed SNPs associated with *a priori* candidate genes, *TT12*, *PIL6*, and *NRT2.7* were linked to significant SNPs for both final proportion germinated and time of maximum germination rate. *TT12* is particularly promising for further study because it was important for more than one germination trait, had a significant SNP within it in both the candidate gene and whole-genome analyses, and had a function that could be clearly related to both light and nutrients. Mutant *TT12* alleles affect germination by altering seed coat permeability to both light and nutrients (Debeaujon *et al.* 2000).

### Comparison with prior studies

#### QTL studies:

Two QTL studies, using the Bay-0×Sha RIL set (Laserna *et al.* 2008; Meng *et al.* 2008), examined the effects of different environments on *A. thaliana* germination. Six QTL were associated with total germination in the dark at 6° (Meng *et al.* 2008), and three were associated with total germination after a red-light pulse (Laserna *et al.* 2008). Between the two studies, two QTL collocated. Because the physical locations of the Bay-0×Sha markers are known (Loudet *et al.* 2002), we were able to estimate that seven of our significant SNPs fell within these two peaks (Table 7). Additionally, three SNPs collocated with two more QTL identified by Meng *et al.* (2008) and two SNPs collated with another QTL identified by Laserna *et al.* (2008). One of the QTL identified by both Laserna *et al.* (2008) and Meng *et al.* (2008) collocated with *PHYB* (At2g18790) and *PIL5* (At2g20180), which were candidate genes in both studies because both are involved in the light-regulated phytochrome pathways and affect *A. thaliana* germination (Oh *et al.* 2004). However, the intervals under these QTL peaks are very large and *PIL5* lies over 1500 kb from the SNPs we identified, with *PHYB* even more distant; therefore, the four SNPs are likely linked to a different gene or genes. No candidate genes in either study collocated with any other QTL or SNPs.

**Table 7:** Meng QTL and Laserna QTL and the significant SNPs that collocate with them

Meng QTL	Laserna QTL	SNP
*CDG-2*	*DOG-10*	Chr2:6351897
*CDG-2*	*DOG-10*	Chr2:8960447
*CDG-2*	*DOG-10*	Chr2:10297188
*CDG-2*	*DOG-10*	Chr2:10297285
*CDG-4*	*NA*	Chr3:17718905
*CDG-3*	*NA*	Chr4:9533814
*CDG-3*	*NA*	Chr4:10089582
*NA*	*DOG-12*	Chr4:12776709
*NA*	*DOG-12*	Chr4:13491707
*CDG-5*	*DOG-11*	Chr5:15976193
*CDG-5*	*DOG-11*	Chr5:16425024
*CDG-5*	*DOG-11*	Chr5:17435459

*NA* indicates that no QTL collocates.

#### GWA studies:

Atwell *et al.* (2010) measured six phenotypes directly related to germination and dormancy: time to 50% germination after two different storage conditions and percent of (nondormant) seeds germinated after 1 wk under four conditions (in the dark at 4° and under 16-hr days at 10°, 16°, and 22°). They reported 10 genes that were plausible germination-related or dormancy-related genes within 20 kb of the most significant SNPs. We did not identify any of the same SNPs or genes, and no SNP we identified was within 20 kb of their SNPs. At best, four SNPs we identified were within 500 kb of their SNPs.

There are at least three possible reasons why we found different significant SNPs from Atwell *et al.* (2010). First, the 100 accessions used in our study overlapped with at most 51 of their 199 accessions (not all of their accessions were used in each of their treatments), so the responses of the accessions we did not have in common could have produced different results. Second, their experimental conditions and measured phenotypes were different from those we measured. Finally, our analyses were different. While we used the MTMM method and were interested in potential G×E interactions, Atwell *et al.* (2010) used EMMAX and were not expressly asking G×E questions. Our finding of fewer and different SNPs using MTMM is not unique. Data for an earlier GWAS study using EMMAX found 92 significant SNPs across environments (Li *et al.* 2010). When that data were re-analyzed using the MTMM method, only 41 SNPs were found in the nine models tested and, based on the information provided in the publications, only nine SNPs were shared between the two studies (Li *et al.* 2010; Korte *et al.* 2012).

## Conclusions

Natural variation in total germination in *A. thaliana* is associated with light and nutrient effects. Although we identified several genomic regions that influence differences in this trait, we likely identified only a subset of the chromosomal regions involved in responses to environmental heterogeneity. To fully understand the role of natural variation in germination timing and cuing, we must study germination using a large number of wild accessions under a large variety of ecologically relevant conditions and begin testing the effects the genetic variants discovered by GWAS have on germination under natural and controlled conditions.

## Supplementary Material

Supporting Information
