# Supplementary material for: Association Mapping of Germination Traits in Arabidopsis thaliana Under Light and Nutrient Treatments: Searching for G×E Effects
Source: G3 (Bethesda). 2014 Jun 5;4(8):1465–78. doi: 10.1534/g3.114.012427 (PMC4132177; doi:10.1534/g3.114.012427)
Supplement: Supporting Information [file supp_4_8_1465__index.html]

Association Mapping of Germination Traits in Arabidopsis thaliana Under Light and Nutrient Treatments: Searching for G×E Effects — Supporting Information 

# Association Mapping of Germination Traits in *Arabidopsis thaliana* Under Light and Nutrient Treatments: Searching for G×E Effects

## Supporting Information for Morrison and Linder, 2014

**Files in this Data Supplement:**

- Supporting Information - Files S1-S9 and Tables S1-S7 (PDF, 194 KB)
- Table S1 - The 100 spring-germinating accessions used in this study. (PDF, 86 KB)
- Table S2 - *A priori* candidate genes and the environmental factor to which they are known to respond. (PDF, 147 KB)
- Table S3 - Genes considered linked to significant SNPs for the FPG phenotype (see manuscript for details), the SNP(s) they are linked to, and model in which the significant SNP was found. (PDF, 90 KB)
- Table S4 - Genes considered linked to significant SNPs for the TMAX phenotype (see manuscript for details), the position of the SNP(s) they are linked to and model in which the significant SNP was found. (PDF, 95 KB)
- Table S5 - Enrichment (or lack thereof) for SNPs linked to candidate genes in the top 50, 100, etc. SNPs for FPC. (PDF, 84 KB)
- Table S6 - Enrichment (or lack thereof) for SNPs linked to candidate genes in the top 50, 100, etc. SNPs for TMAX. (PDF, 84 KB)
- Table S7 - Genes considered linked to significant reaction norm SNPs (see manuscript for details), the SNP(s) they are linked to, and model in which the significant SNP was found. (PDF, 89 KB)
- File S1 - Supporting References (PDF, 115 KB)
- File S2 - Raw p-values from the MTMM test of FPG. (.zip, 11 MB)
- File S3 - Raw p-values from the MTMM test of TMAX. (.zip, 11 MB)
- File S4 - Raw p-values from EMMA test of FPG. (.zip, 6 MB)
- File S5 - Raw p-values from EMMA test of TMAX. (.zip, 6 MB)
- File S6 - Number and proportion of seeds germinated for each replicate at each time point measured. (.txt, 504 KB)
- File S7 - TMAX and maximum germination rate phenotype data. (.txt, 129 KB)
- File S8 - Each accession's SNP genotype from the Nordborg data set. (.zip, 3 MB)
- File S9 - SNP number, basepair, and chromosome information. (.zip, 1 MB)
