# Supplementary material for: Association Mapping of Germination Traits in Arabidopsis thaliana Under Light and Nutrient Treatments: Searching for G×E Effects
Source: G3 (Bethesda). 2014 Jun 5;4(8):1465–78. doi: 10.1534/g3.114.012427 (PMC4132177; doi:10.1534/g3.114.012427)
Supplement: Supporting Information [file supp_g3.114.012427_012427SI.pdf]

G3: Genes | Genomes | Genetics

Association Mapping of Germination Traits in *Arabidopsis thaliana* under Light and Nutrient Treatments: Searching for G x E Effects.

Ginnie D. Morrison*[1] and C. Randal Linder*

*Department of Integrative Biology, University of Texas at Austin, Austin, TX 78712

[1]Corresponding Author: present address, 204 Curtis Hall, University of Missouri Columbia Missouri 65201. Email

morrisong@missouri.edu.

DOI: 10.1534/g3.114.012427

Files S1-S9

Available for download at http://www.g3journal.org/lookup/suppl/doi:10.1534/g3.114.012427/-/DC1

File S1 Supporting References

File S2 Raw p-values from the MTMM test of FPG

File S3 Raw p-values from the MTMM test of TMAX

File S4 Raw p-values from EMMA test of FPG

File S5 Raw p-values from EMMA test of TMAX

File S6 Number and proportion of seeds germinated for each replicate at each time point measured

File S7 TMAX and maximum germination rate phenotype data

File S8 Each accession’s SNP genotype from the Nordborg data set

File S9 SNP number, basepair, and chromosome information

Table S1 The 100 spring-germinating accessions used in this study

Accession[a]	Germplasm[a]	Latitude[b]	Longitude[b]
Aa-0	CS6600	50.9167 N	9.57073 E
Ag-0	CS22630	45 N	1.3 E
Ak-1	CS6602	48.0683 N	7.62551 E
Alc-0	CS1656	40.31 N	3.22 W
ALL1-2	CS76089	45.2667 N	1.48333 E
Alst-1	CS22550	54.8 N	2.4333 W
Amel-1	CS22526	53.448 N	5.73 E
An-1	CS22626	51.2167 N	4.4 E
Ang-0	CS6605	50.3 N	5.3 E
Baa-1	CS22529	51.3333 N	6.1 E
Bch-1	CS6609	49.5166 N	9.3166 E
Bd-0	CS6612	52.4584 N	13.287 E
Boot-1	CS22551	54.4 N	3.2667 W
Bor-1	CS22590	49.4013 N	16.2326 E
Bor-4	CS22591	49.4013 N	16.2326 E
Bsch-0	CS6630	50.0167 N	8.6667 E
Bsch-2	CS6631	50.0167 N	8.6667 E
Chat-1	CS22521	48.0717 N	1.33867 E
CIBC-17	CS22603	51.4083 N	0.6383 W
CIBC-5	CS22602	51.4083 N	0.6383 W
Cvi-0	CS22614	15.1111 N	23.6167 W
Db-0	CS6677	50.3055 N	8.324 E
Dr-0	CS6684	51.051 N	13.7336 E
Dra-2	CS6687	49.4167 N	16.2667E
Ei-2	CS22616	50.3 N	6.3 E
El-0	CS6694	51.5105 N	9.68253 E
Ema-1	CS6923	51.3 N	0.5 E
Fei-0	CS22645	40.5 N	8.32 W
Ga-0	CS22634	50.3 N	8 E
Gel-1	CS22533	51.0167 N	5.86667 E
Gie-0	CS6720	50.584 N	8.67825 E
Gu-0	CS22617	50.3 N	8 E
Gy-0	CS22631	49 N	2 E
H55	CS923	49 N	15 E
Hey-1	CS22534	51.25 N	5.9 E
Hl-3	CS6904	52.1444 N	9.37827 E

HR-10	CS22597	51.4083 N	0.6383 W
HR-5	CS22596	51.4083 N	0.6383 W
Is-1	CS6906	50.5 N	7.5 E
Kin-0	CS22654	44.46 N	85.37 W
Kz-1	CS22606	49.5 N	73.1 E
Kz-9	CS22607	49.5 N	73.1 E
Li-2:1	CS6772	50.3833 N	8.0666 E
LI-OF-095	CS76165	40.7777 N	72.9069 W
LL-0	CS22650	41.59 N	2.49 E
Lm-2	CS1345	48 N	0.5 E
Lp2-2	CS22594	49.38 N	16.81 E
Lp2-6	CS22595	49.38 N	16.81 E
Lz-0	CS22615	46 N	3.3 E
Me-0	CS1364	51.9183 N	10.1138 E
Mh-0	CS6792	50.95 N	7.5 E
MIB-15	CS76181	47.3833 N	5.31667 E
MIB-22	CS76182	47.3833 N	5.31667 E
MIB-28	CS76183	47.3833 N	5.31667 E
MIB-84	CS76184	47.3833 N	5.31667 E

[a]From TAIR (www.arabidopsis.org/index.jsp).

[b]Nordborg dataset (http://papya.usc.edu/2010/data/250k-data-version-3.05).

Table S2 ***A priori* candidate genes and the environmental factor to which they are known to respond.** Genes in bold have a known role in flowering time or vernalization.

Gene Name[a]	Abbreviated Name[a]	Source	Environmental Factor[b]
AT1G03120	ATRAB28	(Atwell *et al.* 2010)	General
AT1G05010	EFE	(Atwell *et al.* 2010)	General
AT1G09950	RAS1	(Ren *et al.* 2010)	General
**AT1G12610**	**DDF1**	(Atwell *et al.* 2010)	General
**AT1G14920**	**GAI**	(Lee *et al.* 2002)	General
AT1G15550	GA3ox1	(Kucera *et al.* 2005)	General
AT1G24260	SEPALLATA3	(Atwell *et al.* 2010)	General
AT1G28560	SRD2	(Atwell *et al.* 2010)	General
AT1G30040	GA2ox2	(Cadman *et al.* 2006)	General
AT1G34790	TT1	(Debeaujon *et al.* 2000)	General
AT1G43620	TT15	(Debeaujon *et al.* 2000)	General
AT1G49040	SCD1	(Atwell *et al.* 2010)	General
**AT1G49480**	**RTV1**	(Atwell *et al.* 2010)	General
**AT1G52340**	**ABA2**	(Koornneef and Jorna 1982)	General
AT1G66350	RGL1	(Lee *et al.* 2002)	General
AT1G72560	PSD	(Atwell *et al.* 2010)	General
AT1G72770	HAB1	(Atwell *et al.* 2010)	General
AT1G72830	HAP2C	(Atwell *et al.* 2010)	General
AT1G78240	TSD2	(Atwell *et al.* 2010)	General
AT1G78390	NCED9	(Cadman *et al.* 2006)	General
AT1G80340	GA3ox2	(Kucera *et al.* 2005)	General
AT2G04240	XERICO	(Zentella *et al.* 2007)	General
**AT2G06210**	**ELF8**	(Atwell *et al.* 2010)	General
AT2G19560	EER5	(Atwell *et al.* 2010)	General
AT2G20000	HBT	(Atwell *et al.* 2010)	General
AT2G25170	PKL	(Atwell *et al.* 2010)	General
AT2G27380	ATEPR1	(Atwell *et al.* 2010)	General
AT2G33830		(Atwell *et al.* 2010)	General
AT2G34900	IMB1	(Duque and Chua 2003)	General
AT2G36270	ABI5	(Finkelstein 1994)	General
**AT2G39810**	**HOS1**	(Atwell *et al.* 2010)	General
AT2G40220	ABI4	(Finkelstein 1994)	General
AT2G42830	SHP2	(Atwell *et al.* 2010)	General
**AT2G44950**	**RDO4/HUB1**	(Liu *et al.* 2007)	General
**AT2G45660**	**AGL20**	(Atwell *et al.* 2010)	General

AT3G03450	RGL2	(Lee *et al.* 2002)	General
AT3G05120	GID1	(Griffiths *et al.* 2006)	General
AT3G05890	RCI2B	(Atwell *et al.* 2010)	General
AT3G11440	ATMYB65	(Atwell *et al.* 2010)	General
**AT3G11540**	**SPY**	(Jacobsen and Olszewski 1993)	General
**AT3G20780**	**ATTOP6B**	(Atwell *et al.* 2010)	General
AT3G24220	NCED6	(Cadman *et al.* 2006)	General
**AT3G24440**	**VRN5**	(Atwell *et al.* 2010)	General
AT3G24650	ABI3	(Koornneef *et al.* 1989)	General
AT3G26120	TEL1	(Atwell *et al.* 2010)	General
AT3G54810	BME3	(Atwell *et al.* 2010)	General
AT3G54990	SMZ	(Atwell *et al.* 2010)	General
AT3G55120	TT5	(Debeaujon *et al.* 2000)	General
AT3G59030	TT12	(Debeaujon *et al.* 2000)	General
AT3G63010	ATGID1B	(Atwell *et al.* 2010)	General
**AT4G02020**	**EZA1**	(Atwell *et al.* 2010)	General
AT4G02570	ATCUL1	(Atwell *et al.* 2010)	General
**AT4G02780**	**GA1**	(Raz *et al.* 2001)	General
**AT4G16280**	**FCA**	(Atwell *et al.* 2010)	General
AT4G18660	sim DOG1	(Atwell *et al.* 2010)	General
AT4G24210	SLY1	(Steber *et al.* 1998)	General
**AT4G24540**	**AGL24**	(Atwell *et al.* 2010)	General
AT4G24620	PGI1	(Atwell *et al.* 2010)	General
AT4G25140	OLEO1	(Atwell *et al.* 2010)	General
**AT4G25530**	**FWA**	(Atwell *et al.* 2010)	General
AT4G26080	ABI1	(Atwell *et al.* 2010)	General
AT4G33280	sim VRN1	(Atwell *et al.* 2010)	General
AT4G39850	COMATOSE	(Russell *et al.* 2000)	General
AT5G01560		(Atwell *et al.* 2010)	General
AT5G02310	PRT6	(Holman *et al.* 2009)	General
AT5G04040	SDP1	(Atwell *et al.* 2010)	General
AT5G07190	ATS3	(Atwell *et al.* 2010)	General
AT5G07280	EMS1	(Atwell *et al.* 2010)	General
AT5G09810	ACT7	(Atwell *et al.* 2010)	General
AT5G09820	TT8	(Debeaujon *et al.* 2000)	General
**AT5G10140**	**FLC**	(Atwell *et al.* 2010)	General
AT5G13790	AGL15	(Atwell *et al.* 2010)	General
AT5G13930	TT4	(Debeaujon *et al.* 2000)	General
AT5G14750	WER1	(Atwell *et al.* 2010)	General

AT5G15100	PIN8	(Atwell *et al.* 2010)	General
**AT5G16320**	**FRL1**	(Atwell *et al.* 2010)	General
**AT5G23150**	**HUA2**	(Atwell *et al.* 2010)	General
AT5G24520	TTG1	(Debeaujon *et al.* 2000)	General
AT5G24630	BIN4	(Atwell *et al.* 2010)	General
AT5G27320	GID1	(Griffiths *et al.* 2006)	General
AT5G35550	TT2	(Debeaujon *et al.* 2000)	General
AT5G42800	TT3	(Debeaujon *et al.* 2000)	General
AT5G45830	DOG1	(Bentsink *et al.* 2006)	General
AT5G47010	LBA1	(Atwell *et al.* 2010)	General
AT5G48100	TT10	(Debeaujon *et al.* 2000)	General
**AT5G57380**	**VIN3**	(Atwell *et al.* 2010)	General
AT5G59710	VIP2	(Atwell *et al.* 2010)	General
**AT5G61850**	**LFY**	(Atwell *et al.* 2010)	General
AT5G62000	ARF2	(Atwell *et al.* 2010)	General
AT5G64210	AOX2	(Atwell *et al.* 2010)	General
AT5G65420	CYCD4;1	(Atwell *et al.* 2010)	General
AT5G67030	ABA1	(Koornneef *et al.* 1989)	General
AT1G01060	LHY	(Atwell *et al.* 2010)	Light
AT1G03790	SOM	(Kim *et al.* 2008)	Light
AT1G09530	PIF3	(Martinez-Garcia *et al.* 2000)	Light
AT1G09570	PHYA	(Shinomura *et al.* 1994)	Light
AT1G14280	PKS2	(Atwell *et al.* 2010)	Light
AT1G52830	IAA6	(Atwell *et al.* 2010)	Light
AT1G53090	SPA4	(Atwell *et al.* 2010)	Light
**AT1G65480**	**FT**	(Atwell *et al.* 2010)	Light
AT1G70940	PIN3	(Atwell *et al.* 2010)	Light
AT1G80730	ZFP1	(Atwell *et al.* 2010)	Light
AT2G01570	RGA	(Dill and Sun 2001)	Light
AT2G18790	PHYB	(Shinomura *et al.* 1994)	Light
AT2G20180	PIL5	(Oh *et al.* 2004)	Light
AT2G32250	FRS2	(Atwell *et al.* 2010)	Light
AT2G37678	FHY1	(Atwell *et al.* 2010)	Light
**AT2G40080**	**ELF4**	(Atwell *et al.* 2010)	Light
AT2G42260	UVI4	(Atwell *et al.* 2010)	Light
AT3G07650	COL9	(Atwell *et al.* 2010)	Light
AT3G09150	HY2	(Atwell *et al.* 2010)	Light
AT3G19820	DWF1	(Atwell *et al.* 2010)	Light
AT3G22380	TIC	(Atwell *et al.* 2010)	Light

AT3G59060	PIL6	(Atwell *et al.* 2010)	Light
**AT4G02560**	**LD**	(Atwell *et al.* 2010)	Light
AT4G03400	DFL2	(Atwell *et al.* 2010)	Light
AT4G11110	SPA2	(Atwell *et al.* 2010)	Light
AT4G16250	PHYD	(Aukerman *et al.* 1997)	Light
AT4G18130	PHYE	(Hennig *et al.* 2002)	Light
AT4G19990	FRS1	(Atwell *et al.* 2010)	Light
AT4G36930	SPT	(Penfield *et al.* 2005)	Light
AT4G37580	HLS1	(Atwell *et al.* 2010)	Light
AT5G25220	KNAT3	(Atwell *et al.* 2010)	Light
AT5G54510	DFL1	(Atwell *et al.* 2010)	Light
AT5G58960	GIL1	(Atwell *et al.* 2010)	Light
**AT5G61380**	**TOC1**	(Atwell *et al.* 2010)	Light
**AT5G62640**	**ELF5**	(Atwell *et al.* 2010)	Light
AT5G64330	NPH3	(Atwell *et al.* 2010)	Light
AT1G12110	NRT1.1	(Alboresi *et al.* 2005)	Nutrient
AT1G37130	NIA2	(Finch-Savage *et al.* 2007)	Nutrient
AT1G77760	NIA1	(Finch-Savage *et al.* 2007)	Nutrient
AT5G14570	NRT2.7	(Chopin *et al.* 2007)	Nutrient

[a]Gene names are from TAIR 10 (www.arabidopsis.org/index.jsp).

[b]General = not known to respond specifically light or nutrient cues. Light = light responsive or in light signaling pathway. Nutrient = responsive to nutrient levels.

Table S3 Genes considered linked to significant SNPs for the FPG phenotype (see manuscript for details), the SNP(s) they are linked to, and model in which the significant SNP was found. Names, descriptions, expression, and GO information from TAIR. Genes in bold are also significant for time of maximum germination rate.

Gene[a]	Name	SNP	Model(s)	Description	Expressed[b]	GO Biological Process
**AT1G08660**	MGP2	Chr1:2757164, Chr1:2759471, Chr1:2763016, Chr1:2765047	Full-Light/Low, Full-Light/High, Full		y	metabolic process, microtubule nucleation
**AT1G08670**		Chr1:2757164, Chr1:2759471, Chr1:2763016, Chr1:2765047	Full-Light/Low, Full-Light/High, Full		n	iron ion transport, nitrate transport, response to nitrate
**AT1G08680**	ZIGA4	Chr1:2759471, Chr1:2763016, Chr1:2765047, Chr1:2770350	Full-Light/Low, Full-Light/High, Full		y	protein autophosphorylation, regulation of ARF GTPase activity
**AT1G08695**	SCRL3	Chr1:2765047, Chr1:2770350	Full-Light/Low, Full-Light/High, Full		n	signal transduction
**AT1G08700**	PS1	Chr1:2765047, Chr1:2770350	Full-Light/Low, Full-Light/High, Full		y	calcium-mediated signaling, intracellular signal transduction, metabolic process
AT1G29750	RKF1	Chr1:10419017	Full-Light/Low		y	oligopeptide transport, protein phosphorylation, transmembrane receptor protein tyrosine kinase signaling pathway
**AT2G24210**	TPS10	Chr2:10297188, Chr2:10297285	Full-Light/Low		y	meristem development, metabolic process, monoterpenoid biosynthetic process,

						response to jasmonic acid stimulus, response to wounding
**AT2G24220**	PUP5	Chr2:10297188	Full-Light/Low		y	nucleobase-containing compound transport
**AT2G24230**		Chr2:10297188	Full-Light/Low		y	protein phosphorylation, transmembrane receptor protein tyrosine kinase signaling pathway
AT2G42290		Chr2:17620611	Full-Light/Low		y	protein phosphorylation, transmembrane receptor protein tyrosine kinase signaling pathway
AT4G08685	SAH7	Chr4:5556326	Full-Light/Low		y	Golgi organization, biological_process, calcium ion transport, cell wall biogenesis, cysteine biosynthetic process, response to salt stress
AT4G08690		Chr4:5556326	Full-Light/Low		y	cell wall biogenesis, transport
AT4G08691		Chr4:5556326	Full-Light/Low	unknown	n	
**AT4G15450**		Chr4:8843014	Full-Light/High	Senescence/dehydration-associated protein-related	n	
**AT4G15460**		Chr4:8843014	Full-Light/High	glycine-rich protein	n	
AT4G16930		Chr4:9533814	Full-Light/High		n	defense response, signal transduction
AT4G16940		Chr4:9533814	Full-Light/High		n	defense response, signal transduction
AT4G26800		Chr4:13491707	Full-Light/Low	Pentatricopeptide repeat superfamily protein		
AT4G26810		Chr4:13491707	Full-Light/Low	SWIB/MDM2 domain superfamily protein	n	
**AT5G28680**	ANX2	Chr5:10723903	Dark/Low		y	protein phosphorylation,

**AT5G28690**		Chr5:10723903	Dark/Low	unknown	n	
**AT5G39880**		Chr5:15976193	Full-Light/Low	unknown	y	
**AT5G39890**		Chr5:15976193	Full-Light/Low		y	cell wall macromolecule metabolic process, oxidation-reduction process, regulation of hydrogen peroxide metabolic process, response to hypoxia, salicylic acid mediated signaling pathway
**AT5G39895**	pre-tRNA	Chr5:15976193	Full-Light/Low	pre-Ala	n	
**AT5G39900**		Chr5:15976193	Full-Light/Low	Small GTP-binding protein	y	
**AT5G39910**		Chr5:15976193	Full-Light/Low	Pectin lyase-like superfamily protein	n	carbohydrate metabolic process

[a]TAIR gene identifier

[b] y = gene is expressed in the seed or embryo, n = not known to be expressed in embryo or seed.

Table S4 Genes considered linked to significant SNPs for the TMAX phenotype (see manuscript for details), the position of the SNP(s) they are linked to and model in which the significant SNP was found. Names, descriptions, expression, and GO information from TAIR. Genes in bold are also significant for FPG.

Gene[a]	Name	SNP	Model(s)	Description	Expressed[b]	GO Biological Process
**AT1G08660**	MGP2	Chr1:2757164, Chr1:2763016, Chr1:2765047	Full-Light/Low, Full-Light/High, Full		y	metabolic process, microtubule nucleation
**AT1G08670**		Chr1:2757164, Chr1:2763016, Chr1:2765047	Full-Light/Low, Full-Light/High, Full		n	iron ion transport, nitrate transport, response to nitrate
**AT1G08680**	ZIGA4	Chr1:2763016, Chr1:2765047, Chr1:2770350	Full-Light/Low, Full-Light/High, Full		y	protein autophosphorylation, regulation of ARF GTPase activity
**AT1G08695**	SCRL3	Chr1:2765047, Chr1:2770350	Full-Light/Low, Full-Light/High, Full		n	signal transduction
**AT1G08700**	PS1	Chr1:2765047, Chr1:2770350	Full-Light/Low, Full-Light/High, Full		y	calcium-mediated signaling, intracellular signal transduction, metabolic process
AT1G12010		Chr1:4058155	Full-Light/Low		n	cellular response to fatty acid, ethylene biosynthetic process, oxidation-reduction process
AT1G12013	SNOR111	Chr1:4058155	Full-Light/Low		n	rRNA modification
AT1G12015		Chr1:4058155	Full-Light/Low		n	rRNA modification
AT1G29750	RKF1	Chr1:10419017	Full-Light/High		y	oligopeptide transport, protein phosphorylation, transmembrane receptor protein tyrosine kinase signaling pathway

AT1G50010	TUA2	Chr1:18526664	Dark/Low		y	GTP catabolic process, microtubule-based movement, microtubule-based process, protein polymerization, response to salt stress
AT1G50020		Chr1:18526664	Dark/Low	unknown	y	
AT1G50030	TOR	Chr1:18526664	Dark/Low		y	actin nucleation, cell adhesion, cell division, cell wall organization, cytokinesis by cell plate formation, embryo development, embryo development ending in seed dormancy, embryonic pattern specification, meiotic DNA double-strand break formation, meiotic chromosome segregation, negative regulation of autophagy, organ morphogenesis, positive regulation of cell growth, positive regulation of embryonic development, positive regulation of organelle organization, positive regulation of rRNA processing, positive regulation of transcription, DNA-dependent, post-embryonic development, primary shoot apical meristem specification, rRNA transcription, reciprocal meiotic recombination, regulation of cell differentiation, regulation of chromosome organization, root hair cell differentiation, seed development, seed maturation, sister chromatid cohesion, tissue development, toxin catabolic process, trichome morphogenesis, vegetative to reproductive phase transition of meristem
AT2G14800		Chr2:6351897	Full-Light/High		y	reciprocal meiotic recombination, synapsis
AT2G20780		Chr2:8960447	Full-Light/High	Major facilitator superfamily protein	y	transmembrane transport, transport
AT2G20784		Chr2:8960447	Full-Light/High	unknown	n	
AT2G20790		Chr2:8960447	Full-Light/High		y	intracellular protein transport, vesicle-mediated transport

AT2G20800	NDB4	Chr2:8960447	Full-Light/High		n	oxidation-reduction process
AT2G20805		Chr2:8960447	Full-Light/High	unknown	n	
AT2G20810	GAUT10	Chr2:8960447	Full-Light/High		y	carbohydrate biosynthetic process
AT2G20815		Chr2:8960447	Full-Light/High	unknown	n	
AT2G20820		Chr2:8960447	Full-Light/High		y	photorespiration
AT2G20825	ULT2	Chr2:8960447	Full-Light/High		y	
AT2G20830		Chr2:8960447	Full-Light/High		y	metabolic process
**AT2G24210**	TPS10	Chr2:10297188	Full-Light/Low		y	meristem development, metabolic process, monoterpenoid biosynthetic process, response to jasmonic acid stimulus, response to wounding
**AT2G24220**	PUP5	Chr2:10297188	Full-Light/Low		y	nucleobase-containing compound transport
**AT2G24230**		Chr2:10297188	Full-Light/Low		y	protein phosphorylation, transmembrane receptor protein tyrosine kinase signaling pathway
**AT2G42290**		Chr2:17620611	Full-Light/High, Full-Light/Low		y	protein phosphorylation, transmembrane receptor protein tyrosine kinase signaling pathway
AT3G14330		Chr3:4786505	Full-Light/Low	Tetratricopeptide repeat-like superfamily protein	y	mRNA modification
AT3G14340		Chr3:4786505	Full-Light/Low	unknown	y	
AT3G14350	SRF7	Chr3:4786505	Full-Light/Low		y	protein phosphorylation, transmembrane receptor protein tyrosine kinase signaling pathway
AT3G47990	SIS3	Chr3:17718905	Full-Light/High		y	glucuronoxylan metabolic process, protein ubiquitination, response to high light intensity, response to hydrogen peroxide, sugar mediated signaling pathway, xylan biosynthetic process
AT3G48000	ALDH2	Chr3:17718905	Full-Light/High		y	metabolic process, oxidation-reduction process, response to cadmium ion

AT3G48010	CNGC16	Chr3:17718905	Full-Light/High		n	ion transport, transmembrane transport
**AT3G59020**		Chr3:21818882	Full-Light/High		y	intracellular protein transport, protein import into nucleus, docking
**AT3G59030**	TT12	Chr3:21818882	Full-Light/High		y	drug transmembrane transport, maintenance of seed dormancy, proanthocyanidin biosynthetic process, purine nucleobase transport, transmembrane transport
**AT3G59040**		Chr3:21818882	Full-Light/High	Tetratricopeptide repeat-like superfamily protein	y	chloroplast organization, pentose-phosphate shunt, rRNA processing, tRNA metabolic process
AT4G12240		Chr4:7287800	Full-Light/High		y	regulation of transcription, DNA-dependent
AT4G12250	GAE5	Chr4:7287800	Full-Light/High		y	carbohydrate metabolic process, cellular metabolic process, cellular response to phosphate starvation, cellular response to water deprivation, galactolipid biosynthetic process, nucleotide-sugar metabolic process
**AT4G13180**		Chr4:7657583	Full-Light/High		y	metabolic process, response to arsenic-containing substance
AT4G15450		Chr4:8841131, Chr4:8843014, Chr4:8843150	Full-Light/High, Full-Light/Low	Senescence/dehydration-associated protein-related	n	
AT4G15460		Chr4:8841131, Chr4:8843014, Chr4:8843150	Full-Light/High, Full-Light/Low	glycine-rich protein	n	
AT4G24790		Chr4:12776709	Full-Light/High		y	DNA replication
**AT5G28680**	ANX2	Chr5:10723903	Dark/Low		y	protein phosphorylation
**AT5G28690**		Chr5:10723903	Dark/Low	unknown	n	
AT5G41010	NRPB12	Chr5:16425024	Full-Light/Low		y	RNA splicing, via endonucleolytic cleavage and ligation, transcription from RNA polymerase II promoter, transcription, DNA-dependent

AT5G55340		Chr5:22442725	Full-Light/High	membrane bound O-acyl transferase family protein	y	
AT5G55350		Chr5:22442725	Full-Light/High	membrane bound O-acyl transferase family protein	n	
AT5G55360		Chr5:22442725	Full-Light/High	membrane bound O-acyl transferase family protein	n	
AT5G66690	UGT72E2	Chr5:26627873	Full-Light/High		y	Golgi vesicle transport, RNA methylation, cell wall modification, cellulose biosynthetic process, plant-type cell wall organization

[a]TAIR gene identifier

[b] y = gene is expressed in the seed or embryo, n = not known to be expressed in embryo or seed.

Table S5 Enrichment (or lack thereof) for SNPs linked to candidate genes in the top 50, 100, etc. SNPs for FPC. Enrichment was never significant based on 10000 permutations.

	Enrichment					
**Model**	**50**	**100**	**250**	**500**	**1000**	**5000**
Dark/Low	0	0	0	1.18	1.06	0.59
Dark/High	0	0	0.47	0.94	0.94	0.92
Full-Light/Low	0	0	0.47	0.47	0.35	0.87
Full-Light/High	2.39	2.39	1.90	1.42	1.78	1.01
Full	2.39	1.18	0.94	0.47	0.82	0.82
Genotype Only	2.39	1.18	0.47	0.47	0.71	0.80
GxE	0	0	0.47	0.71	1.06	0.78
GxL	0	1.18	0.94	0.94	1.06	1.06
GxN	0	0	0	0.47	0.35	0.61

Table S6 Enrichment (or lack thereof) for SNPs linked to candidate genes in the top 50, 100, etc. SNPs for TMAX. Enrichment was never significant based on 10000 permutations.

	Enrichment					
**Model**	**50**	**100**	**250**	**500**	**1000**	**5000**
Dark/Low	0	0	0	1.18	1.06	0.59
Dark/High	0	0	0.47	0.94	0.94	0.92
Full-Light/Low	0	0	0.47	0.47	0.35	0.87
Full-Light/High	2.39	2.39	1.90	1.42	1.78	1.01
Full	2.39	1.18	0.94	0.47	0.82	0.82
Genotype Only	2.39	1.18	0.47	0.47	0.71	0.80
GxE	0	0	0.47	0.71	1.06	0.78
GxL	0	1.18	0.94	0.94	1.06	1.06
GxN	0	0	0	0.47	0.35	0.61

Table S7 Genes considered linked to significant reaction norm SNPs (see manuscript for details), the SNP(s) they are linked to, and model in which the significant SNP was found. Names, descriptions, expression, and GO information from TAIR.

Gene[a]	Name	SNP	Norm	Description	Expressed[b]	GO Biological Process
AT1G61890		Chr1:22870338	FPG, Nutrient under Full	MATE efflux family protein	y	drug transmembrane transport, jasmonic acid metabolic process, proline transport, response to abscisic acid stimulus, response to chitin, response to jasmonic acid stimulus, response to karrikin, response to salt stress, transmembrane transport
AT2G05755		Chr2:2177432	FPG, Light under Low	Nucleotide/sugar transporter family protein	y	sphingoid biosynthetic process, sterol biosynthetic process
AT2G24210	TPS10	Chr2:10297188	FPG, Nutrient under Full		y	meristem development, metabolic process, monoterpenoid biosynthetic process, response to jasmonic acid stimulus, response to wounding
AT2G24220	PUP5	Chr2:10297188	FPG, Nutrient under Full		y	nucleobase-containing compound transport
AT2G24230		Chr2:10297188	FPG, Nutrient under Full		y	protein phosphorylation, transmembrane receptor protein tyrosine kinase signaling pathway
AT4G18250		Chr4:10089582	TMAX, Light under High	receptor serine/threonine kinase	n	protein phosphorylation
AT5G07315	pre-tRNA	Chr5:2319344	FPG, Light under Low	pre-Tyr	n	translational elongation
AT5G07320	APC3	Chr5:2319344	FPG, Light under Low		y	ATP transport, Golgi localization, actin filament-based movement, mitochondrion localization, peroxisome localization, transmembrane transport, transport
AT5G07322		Chr5:2319344	FPG, Light	other RNA	n	unknown

			under Low			
AT5G07330		Chr5:2319344	FPG, Light under Low		n	unknown
AT5G07340		Chr5:2319344	FPG, Light under Low	Calreticulin family protein	y	protein folding, response to endoplasmic reticulum stress, response to heat, response to high light intensity, response to hydrogen peroxide
AT5G07350	TUDOR1	Chr5:2319344	FPG, Light under Low		y	Golgi vesicle transport, cellulose biosynthetic process, gene silencing by RNA, protein secretion, response to cadmium ion, response to salt stress
AT5G19095	pre-tRNA	Chr5:6399525	FPG, Nutrient under Full	pre-Gly	n	
AT5G39880		Chr5:15976193	FPG, Nutrient under Full	unknown	y	
AT5G39890		Chr5:15976193	FPG, Nutrient under Full		y	cell wall macromolecule metabolic process, oxidation-reduction process, regulation of hydrogen peroxide metabolic process, response to hypoxia, salicylic acid mediated signaling pathway
AT5G39895	pre-tRNA	Chr5:15976193	FPG, Nutrient under Full	pre-Ala	n	
AT5G39900		Chr5:15976193	FPG, Nutrient under Full	Small GTP-binding protein	y	
AT5G39910		Chr5:15976193	FPG, Nutrient under Full	Pectin lyase-like superfamily protein	n	carbohydrate metabolic process

[a]TAIR gene identifier

[b] y = gene is expressed in the seed or embryo, n = not known to be expressed in embryo or seed.
