# Supplementary material for: Association Mapping of Germination Traits in Arabidopsis thaliana Under Light and Nutrient Treatments: Searching for G×E Effects
Source: G3 (Bethesda). 2014 Jun 5;4(8):1465–78. doi: 10.1534/g3.114.012427 (PMC4132177; doi:10.1534/g3.114.012427)
Supplement: Supporting Information [file supp_g3.114.012427_TableS1.pdf]

Table S1   The 100 spring-germinating accessions used in this study

Accession[a]	Germplasm[a]	Latitude[b]	Longitude[b]
Aa-0	CS6600	50.9167 N	9.57073 E
Ag-0	CS22630	45 N	1.3 E
Ak-1	CS6602	48.0683 N	7.62551 E
Alc-0	CS1656	40.31 N	3.22 W
ALL1-2	CS76089	45.2667 N	1.48333 E
Alst-1	CS22550	54.8 N	2.4333 W
Amel-1	CS22526	53.448 N	5.73 E
An-1	CS22626	51.2167 N	4.4 E
Ang-0	CS6605	50.3 N	5.3 E
Baa-1	CS22529	51.3333 N	6.1 E
Bch-1	CS6609	49.5166 N	9.3166 E
Bd-0	CS6612	52.4584 N	13.287 E
Boot-1	CS22551	54.4 N	3.2667 W
Bor-1	CS22590	49.4013 N	16.2326 E
Bor-4	CS22591	49.4013 N	16.2326 E
Bsch-0	CS6630	50.0167 N	8.6667 E
Bsch-2	CS6631	50.0167 N	8.6667 E
Chat-1	CS22521	48.0717 N	1.33867 E
CIBC-17	CS22603	51.4083 N	0.6383 W
CIBC-5	CS22602	51.4083 N	0.6383 W
Cvi-0	CS22614	15.1111 N	23.6167 W
Db-0	CS6677	50.3055 N	8.324 E
Dr-0	CS6684	51.051 N	13.7336 E
Dra-2	CS6687	49.4167 N	16.2667E
Ei-2	CS22616	50.3 N	6.3 E
El-0	CS6694	51.5105 N	9.68253 E
Ema-1	CS6923	51.3 N	0.5 E
Fei-0	CS22645	40.5 N	8.32 W
Ga-0	CS22634	50.3 N	8 E
Gel-1	CS22533	51.0167 N	5.86667 E
Gie-0	CS6720	50.584 N	8.67825 E
Gu-0	CS22617	50.3 N	8 E
Gy-0	CS22631	49 N	2 E
H55	CS923	49 N	15 E
Hey-1	CS22534	51.25 N	5.9 E
Hl-3	CS6904	52.1444 N	9.37827 E

HR-10	CS22597	51.4083 N	0.6383 W
HR-5	CS22596	51.4083 N	0.6383 W
Is-1	CS6906	50.5 N	7.5 E
Kin-0	CS22654	44.46 N	85.37 W
Kz-1	CS22606	49.5 N	73.1 E
Kz-9	CS22607	49.5 N	73.1 E
Li-2:1	CS6772	50.3833 N	8.0666 E
LI-OF-095	CS76165	40.7777 N	72.9069 W
LL-0	CS22650	41.59 N	2.49 E
Lm-2	CS1345	48 N	0.5 E
Lp2-2	CS22594	49.38 N	16.81 E
Lp2-6	CS22595	49.38 N	16.81 E
Lz-0	CS22615	46 N	3.3 E
Me-0	CS1364	51.9183 N	10.1138 E
Mh-0	CS6792	50.95 N	7.5 E
MIB-15	CS76181	47.3833 N	5.31667 E
MIB-22	CS76182	47.3833 N	5.31667 E
MIB-28	CS76183	47.3833 N	5.31667 E
MIB-84	CS76184	47.3833 N	5.31667 E

[a]From TAIR (www.arabidopsis.org/index.jsp).

[b]Nordborg dataset (http://papya.usc.edu/2010/data/250k-data-version-3.05).
