# Supplementary material for: Association Mapping of Germination Traits in Arabidopsis thaliana Under Light and Nutrient Treatments: Searching for G×E Effects
Source: G3 (Bethesda). 2014 Jun 5;4(8):1465–78. doi: 10.1534/g3.114.012427 (PMC4132177; doi:10.1534/g3.114.012427)
Supplement: Supporting Information [file supp_g3.114.012427_TableS2.pdf]

Table S2 ***A priori*** **candidate genes and the environmental factor to which they are known to respond.** Genes in bold have a known role in flowering time or vernalization.

Gene Name[a]	Abbreviated Name[a]	Source	Environmental Factor[b]
AT1G03120	ATRAB28	(Atwell *et al.* 2010)	General
AT1G05010	EFE	(Atwell *et al.* 2010)	General
AT1G09950	RAS1	(Ren *et al.* 2010)	General
**AT1G12610**	**DDF1**	(Atwell *et al.* 2010)	General
**AT1G14920**	**GAI**	(Lee *et al.* 2002)	General
AT1G15550	GA3ox1	(Kucera *et al.* 2005)	General
AT1G24260	SEPALLATA3	(Atwell *et al.* 2010)	General
AT1G28560	SRD2	(Atwell *et al.* 2010)	General
AT1G30040	GA2ox2	(Cadman *et al.* 2006)	General
AT1G34790	TT1	(Debeaujon *et al.* 2000)	General
AT1G43620	TT15	(Debeaujon *et al.* 2000)	General
AT1G49040	SCD1	(Atwell *et al.* 2010)	General
**AT1G49480**	**RTV1**	(Atwell *et al.* 2010)	General
**AT1G52340**	**ABA2**	(Koornneef and Jorna 1982)	General
AT1G66350	RGL1	(Lee *et al.* 2002)	General
AT1G72560	PSD	(Atwell *et al.* 2010)	General
AT1G72770	HAB1	(Atwell *et al.* 2010)	General
AT1G72830	HAP2C	(Atwell *et al.* 2010)	General
AT1G78240	TSD2	(Atwell *et al.* 2010)	General
AT1G78390	NCED9	(Cadman *et al.* 2006)	General
AT1G80340	GA3ox2	(Kucera *et al.* 2005)	General
AT2G04240	XERICO	(Zentella *et al.* 2007)	General
**AT2G06210**	**ELF8**	(Atwell *et al.* 2010)	General
AT2G19560	EER5	(Atwell *et al.* 2010)	General
AT2G20000	HBT	(Atwell *et al.* 2010)	General
AT2G25170	PKL	(Atwell *et al.* 2010)	General
AT2G27380	ATEPR1	(Atwell *et al.* 2010)	General
AT2G33830		(Atwell *et al.* 2010)	General
AT2G34900	IMB1	(Duque and Chua 2003)	General
AT2G36270	ABI5	(Finkelstein 1994)	General
**AT2G39810**	**HOS1**	(Atwell *et al.* 2010)	General
AT2G40220	ABI4	(Finkelstein 1994)	General
AT2G42830	SHP2	(Atwell *et al.* 2010)	General
**AT2G44950**	**RDO4/HUB1**	(Liu *et al.* 2007)	General
**AT2G45660**	**AGL20**	(Atwell *et al.* 2010)	General

AT3G03450	RGL2	(Lee *et al.* 2002)	General
AT3G05120	GID1	(Griffiths *et al.* 2006)	General
AT3G05890	RCI2B	(Atwell *et al.* 2010)	General
AT3G11440	ATMYB65	(Atwell *et al.* 2010)	General
**AT3G11540**	**SPY**	(Jacobsen and Olszewski 1993)	General
**AT3G20780**	**ATTOP6B**	(Atwell *et al.* 2010)	General
AT3G24220	NCED6	(Cadman *et al.* 2006)	General
**AT3G24440**	**VRN5**	(Atwell *et al.* 2010)	General
AT3G24650	ABI3	(Koornneef *et al.* 1989)	General
AT3G26120	TEL1	(Atwell *et al.* 2010)	General
AT3G54810	BME3	(Atwell *et al.* 2010)	General
AT3G54990	SMZ	(Atwell *et al.* 2010)	General
AT3G55120	TT5	(Debeaujon *et al.* 2000)	General
AT3G59030	TT12	(Debeaujon *et al.* 2000)	General
AT3G63010	ATGID1B	(Atwell *et al.* 2010)	General
**AT4G02020**	**EZA1**	(Atwell *et al.* 2010)	General
AT4G02570	ATCUL1	(Atwell *et al.* 2010)	General
**AT4G02780**	**GA1**	(Raz *et al.* 2001)	General
**AT4G16280**	**FCA**	(Atwell *et al.* 2010)	General
AT4G18660	sim DOG1	(Atwell *et al.* 2010)	General
AT4G24210	SLY1	(Steber *et al.* 1998)	General
**AT4G24540**	**AGL24**	(Atwell *et al.* 2010)	General
AT4G24620	PGI1	(Atwell *et al.* 2010)	General
AT4G25140	OLEO1	(Atwell *et al.* 2010)	General
**AT4G25530**	**FWA**	(Atwell *et al.* 2010)	General
AT4G26080	ABI1	(Atwell *et al.* 2010)	General
AT4G33280	sim VRN1	(Atwell *et al.* 2010)	General
AT4G39850	COMATOSE	(Russell *et al.* 2000)	General
AT5G01560		(Atwell *et al.* 2010)	General
AT5G02310	PRT6	(Holman *et al.* 2009)	General
AT5G04040	SDP1	(Atwell *et al.* 2010)	General
AT5G07190	ATS3	(Atwell *et al.* 2010)	General
AT5G07280	EMS1	(Atwell *et al.* 2010)	General
AT5G09810	ACT7	(Atwell *et al.* 2010)	General
AT5G09820	TT8	(Debeaujon *et al.* 2000)	General
**AT5G10140**	**FLC**	(Atwell *et al.* 2010)	General
AT5G13790	AGL15	(Atwell *et al.* 2010)	General
AT5G13930	TT4	(Debeaujon *et al.* 2000)	General
AT5G14750	WER1	(Atwell *et al.* 2010)	General

AT5G15100	PIN8	(Atwell *et al.* 2010)	General
**AT5G16320**	**FRL1**	(Atwell *et al.* 2010)	General
**AT5G23150**	**HUA2**	(Atwell *et al.* 2010)	General
AT5G24520	TTG1	(Debeaujon *et al.* 2000)	General
AT5G24630	BIN4	(Atwell *et al.* 2010)	General
AT5G27320	GID1	(Griffiths *et al.* 2006)	General
AT5G35550	TT2	(Debeaujon *et al.* 2000)	General
AT5G42800	TT3	(Debeaujon *et al.* 2000)	General
AT5G45830	DOG1	(Bentsink *et al.* 2006)	General
AT5G47010	LBA1	(Atwell *et al.* 2010)	General
AT5G48100	TT10	(Debeaujon *et al.* 2000)	General
**AT5G57380**	**VIN3**	(Atwell *et al.* 2010)	General
AT5G59710	VIP2	(Atwell *et al.* 2010)	General
**AT5G61850**	**LFY**	(Atwell *et al.* 2010)	General
AT5G62000	ARF2	(Atwell *et al.* 2010)	General
AT5G64210	AOX2	(Atwell *et al.* 2010)	General
AT5G65420	CYCD4;1	(Atwell *et al.* 2010)	General
AT5G67030	ABA1	(Koornneef *et al*. 1989)	General
AT1G01060	LHY	(Atwell *et al.* 2010)	Light
AT1G03790	SOM	(Kim *et al.* 2008)	Light
AT1G09530	PIF3	(Martinez-Garcia *et al.* 2000)	Light
AT1G09570	PHYA	(Shinomura *et al.* 1994)	Light
AT1G14280	PKS2	(Atwell *et al.* 2010)	Light
AT1G52830	IAA6	(Atwell *et al.* 2010)	Light
AT1G53090	SPA4	(Atwell *et al.* 2010)	Light
**AT1G65480**	**FT**	(Atwell *et al.* 2010)	Light
AT1G70940	PIN3	(Atwell *et al.* 2010)	Light
AT1G80730	ZFP1	(Atwell *et al.* 2010)	Light
AT2G01570	RGA	(Dill and Sun 2001)	Light
AT2G18790	PHYB	(Shinomura *et al.* 1994)	Light
AT2G20180	PIL5	(Oh *et al.* 2004)	Light
AT2G32250	FRS2	(Atwell *et al.* 2010)	Light
AT2G37678	FHY1	(Atwell *et al.* 2010)	Light
**AT2G40080**	**ELF4**	(Atwell *et al.* 2010)	Light
AT2G42260	UVI4	(Atwell *et al.* 2010)	Light
AT3G07650	COL9	(Atwell *et al.* 2010)	Light
AT3G09150	HY2	(Atwell *et al.* 2010)	Light
AT3G19820	DWF1	(Atwell *et al.* 2010)	Light
AT3G22380	TIC	(Atwell *et al.* 2010)	Light

AT3G59060	PIL6	(Atwell *et al.* 2010)	Light
**AT4G02560**	**LD**	(Atwell *et al.* 2010)	Light
AT4G03400	DFL2	(Atwell *et al.* 2010)	Light
AT4G11110	SPA2	(Atwell *et al.* 2010)	Light
AT4G16250	PHYD	(Aukerman *et al.* 1997)	Light
AT4G18130	PHYE	(Hennig *et al.* 2002)	Light
AT4G19990	FRS1	(Atwell *et al.* 2010)	Light
AT4G36930	SPT	(Penfield *et al.* 2005)	Light
AT4G37580	HLS1	(Atwell *et al.* 2010)	Light
AT5G25220	KNAT3	(Atwell *et al.* 2010)	Light
AT5G54510	DFL1	(Atwell *et al.* 2010)	Light
AT5G58960	GIL1	(Atwell *et al.* 2010)	Light
**AT5G61380**	**TOC1**	(Atwell *et al.* 2010)	Light
**AT5G62640**	**ELF5**	(Atwell *et al.* 2010)	Light
AT5G64330	NPH3	(Atwell *et al.* 2010)	Light
AT1G12110	NRT1.1	(Alboresi *et al.* 2005)	Nutrient
AT1G37130	NIA2	(Finch-Savage *et al.* 2007)	Nutrient
AT1G77760	NIA1	(Finch-Savage *et al.* 2007)	Nutrient
AT5G14570	NRT2.7	(Chopin *et al.* 2007)	Nutrient

[a]Gene names are from TAIR 10 (www.arabidopsis.org/index.jsp).

[b]General = not known to respond specifically light or nutrient cues. Light = light responsive or in light signaling pathway. Nutrient = responsive to nutrient levels.
