# Supplementary material for: Association Mapping of Germination Traits in Arabidopsis thaliana Under Light and Nutrient Treatments: Searching for G×E Effects
Source: G3 (Bethesda). 2014 Jun 5;4(8):1465–78. doi: 10.1534/g3.114.012427 (PMC4132177; doi:10.1534/g3.114.012427)
Supplement: Supporting Information [file supp_g3.114.012427_TableS3.pdf]

**Table S3 Genes considered linked to significant SNPs for the FPG phenotype (see manuscript for details), the SNP(s) they are linked to, and model in which the significant SNP was found.** Names, descriptions, expression, and GO information from TAIR. Genes in bold are also significant for time of maximum germination rate.

| Gene[a] | Name | SNP | Model(s) | Description | Expressed[b] | GO Biological Process |
|---|---|---|---|---|---|---|
| **AT1G08660** | MGP2 | Chr1:2757164, Chr1:2759471, Chr1:2763016, Chr1:2765047 | Full-Light/Low, Full-Light/High, Full | | y | metabolic process, microtubule nucleation |
| **AT1G08670** | | Chr1:2757164, Chr1:2759471, Chr1:2763016, Chr1:2765047 | Full-Light/Low, Full-Light/High, Full | | n | iron ion transport, nitrate transport, response to nitrate |
| **AT1G08680** | ZIGA4 | Chr1:2759471, Chr1:2763016, Chr1:2765047, Chr1:2770350 | Full-Light/Low, Full-Light/High, Full | | y | protein autophosphorylation, regulation of ARF GTPase activity |
| **AT1G08695** | SCRL3 | Chr1:2765047, Chr1:2770350 | Full-Light/Low, Full-Light/High, Full | | n | signal transduction |
| **AT1G08700** | PS1 | Chr1:2765047, Chr1:2770350 | Full-Light/Low, Full-Light/High, Full | | y | calcium-mediated signaling, intracellular signal transduction, metabolic process |
| AT1G29750 | RKF1 | Chr1:10419017 | Full-Light/Low | | y | oligopeptide transport, protein phosphorylation, transmembrane receptor protein tyrosine kinase signaling pathway |
| **AT2G24210** | TPS10 | Chr2:10297188, Chr2:10297285 | Full-Light/Low | | y | meristem development, metabolic process, monoterpenoid biosynthetic process, |

| | | | | | | |
|---|---|---|---|---|---|---|
| | | | | | | response to jasmonic acid stimulus, response to wounding |
| **AT2G24220** | PUP5 | Chr2:10297188 | Full-Light/Low | | y | nucleobase-containing compound transport |
| **AT2G24230** | | Chr2:10297188 | Full-Light/Low | | y | protein phosphorylation, transmembrane receptor protein tyrosine kinase signaling pathway |
| AT2G42290 | | Chr2:17620611 | Full-Light/Low | | y | protein phosphorylation, transmembrane receptor protein tyrosine kinase signaling pathway |
| AT4G08685 | SAH7 | Chr4:5556326 | Full-Light/Low | | y | Golgi organization, biological_process, calcium ion transport, cell wall biogenesis, cysteine biosynthetic process, response to salt stress |
| AT4G08690 | | Chr4:5556326 | Full-Light/Low | | y | cell wall biogenesis, transport |
| AT4G08691 | | Chr4:5556326 | Full-Light/Low | unknown | n | |
| **AT4G15450** | | Chr4:8843014 | Full-Light/High | Senescence/dehydration-associated protein-related | n | |
| **AT4G15460** | | Chr4:8843014 | Full-Light/High | glycine-rich protein | n | |
| AT4G16930 | | Chr4:9533814 | Full-Light/High | | n | defense response, signal transduction |
| AT4G16940 | | Chr4:9533814 | Full-Light/High | | n | defense response, signal transduction |
| AT4G26800 | | Chr4:13491707 | Full-Light/Low | Pentatricopeptide repeat superfamily protein | | |
| AT4G26810 | | Chr4:13491707 | Full-Light/Low | SWIB/MDM2 domain superfamily protein | n | |
| **AT5G28680** | ANX2 | Chr5:10723903 | Dark/Low | | y | protein phosphorylation, |

| | | | | | | |
|---|---|---|---|---|---|---|
| **AT5G28690** | | Chr5:10723903 | Dark/Low | unknown | n | |
| **AT5G39880** | | Chr5:15976193 | Full-Light/Low | unknown | y | |
| **AT5G39890** | | Chr5:15976193 | Full-Light/Low | | y | cell wall macromolecule metabolic process, oxidation-reduction process, regulation of hydrogen peroxide metabolic process, response to hypoxia, salicylic acid mediated signaling pathway |
| **AT5G39895** | pre-tRNA | Chr5:15976193 | Full-Light/Low | pre-Ala | n | |
| **AT5G39900** | | Chr5:15976193 | Full-Light/Low | Small GTP-binding protein | y | |
| **AT5G39910** | | Chr5:15976193 | Full-Light/Low | Pectin lyase-like superfamily protein | n | carbohydrate metabolic process |

[a]TAIR gene identifier

[b] y = gene is expressed in the seed or embryo, n = not known to be expressed in embryo or seed.
