# Supplementary material for: Association Mapping of Germination Traits in Arabidopsis thaliana Under Light and Nutrient Treatments: Searching for G×E Effects
Source: G3 (Bethesda). 2014 Jun 5;4(8):1465–78. doi: 10.1534/g3.114.012427 (PMC4132177; doi:10.1534/g3.114.012427)
Supplement: Supporting Information [file supp_g3.114.012427_TableS4.pdf]

Table S4 Genes considered linked to significant SNPs for the TMAX phenotype (see manuscript for details), the position of the SNP(s) they are linked to and model in which the significant SNP was found. Names, descriptions, expression, and GO information from TAIR. Genes in bold are also significant for FPG.

Gene[a]	Name	SNP	Model(s)	Description	Expressed[b]	GO Biological Process
**AT1G08660**	MGP2	Chr1:2757164, Chr1:2763016, Chr1:2765047	Full-Light/Low, Full-Light/High, Full		y	metabolic process, microtubule nucleation
**AT1G08670**		Chr1:2757164, Chr1:2763016, Chr1:2765047	Full-Light/Low, Full-Light/High, Full		n	iron ion transport, nitrate transport, response to nitrate
**AT1G08680**	ZIGA4	Chr1:2763016, Chr1:2765047, Chr1:2770350	Full-Light/Low, Full-Light/High, Full		y	protein autophosphorylation, regulation of ARF GTPase activity
**AT1G08695**	SCRL3	Chr1:2765047, Chr1:2770350	Full-Light/Low, Full-Light/High, Full		n	signal transduction
**AT1G08700**	PS1	Chr1:2765047, Chr1:2770350	Full-Light/Low, Full-Light/High, Full		y	calcium-mediated signaling, intracellular signal transduction, metabolic process
AT1G12010		Chr1:4058155	Full-Light/Low		n	cellular response to fatty acid, ethylene biosynthetic process, oxidation-reduction process
AT1G12013	SNOR111	Chr1:4058155	Full-Light/Low		n	rRNA modification
AT1G12015		Chr1:4058155	Full-Light/Low		n	rRNA modification
AT1G29750	RKF1	Chr1:10419017	Full-Light/High		y	oligopeptide transport, protein phosphorylation, transmembrane receptor protein tyrosine kinase signaling pathway

AT1G50010	TUA2	Chr1:18526664	Dark/Low		y	GTP catabolic process, microtubule-based movement, microtubule-based process, protein polymerization, response to salt stress
AT1G50020		Chr1:18526664	Dark/Low	unknown	y	
AT1G50030	TOR	Chr1:18526664	Dark/Low		y	actin nucleation, cell adhesion, cell division, cell wall organization, cytokinesis by cell plate formation, embryo development, embryo development ending in seed dormancy, embryonic pattern specification, meiotic DNA double-strand break formation, meiotic chromosome segregation, negative regulation of autophagy, organ morphogenesis, positive regulation of cell growth, positive regulation of embryonic development, positive regulation of organelle organization, positive regulation of rRNA processing, positive regulation of transcription, DNA-dependent, post-embryonic development, primary shoot apical meristem specification, rRNA transcription, reciprocal meiotic recombination, regulation of cell differentiation, regulation of chromosome organization, root hair cell differentiation, seed development, seed maturation, sister chromatid cohesion, tissue development, toxin catabolic process, trichome morphogenesis, vegetative to reproductive phase transition of meristem
AT2G14800		Chr2:6351897	Full-Light/High		y	reciprocal meiotic recombination, synapsis
AT2G20780		Chr2:8960447	Full-Light/High	Major facilitator superfamily protein	y	transmembrane transport, transport
AT2G20784		Chr2:8960447	Full-Light/High	unknown	n	
AT2G20790		Chr2:8960447	Full-Light/High		y	intracellular protein transport, vesicle-mediated transport

AT2G20800	NDB4	Chr2:8960447	Full-Light/High		n	oxidation-reduction process
AT2G20805		Chr2:8960447	Full-Light/High	unknown	n	
AT2G20810	GAUT10	Chr2:8960447	Full-Light/High		y	carbohydrate biosynthetic process
AT2G20815		Chr2:8960447	Full-Light/High	unknown	n	
AT2G20820		Chr2:8960447	Full-Light/High		y	photorespiration
AT2G20825	ULT2	Chr2:8960447	Full-Light/High		y	
AT2G20830		Chr2:8960447	Full-Light/High		y	metabolic process
**AT2G24210**	TPS10	Chr2:10297188	Full-Light/Low		y	meristem development, metabolic process, monoterpenoid biosynthetic process, response to jasmonic acid stimulus, response to wounding
**AT2G24220**	PUP5	Chr2:10297188	Full-Light/Low		y	nucleobase-containing compound transport
**AT2G24230**		Chr2:10297188	Full-Light/Low		y	protein phosphorylation, transmembrane receptor protein tyrosine kinase signaling pathway
**AT2G42290**		Chr2:17620611	Full-Light/High, Full-Light/Low		y	protein phosphorylation, transmembrane receptor protein tyrosine kinase signaling pathway
AT3G14330		Chr3:4786505	Full-Light/Low	Tetratricopeptide repeat-like superfamily protein	y	mRNA modification
AT3G14340		Chr3:4786505	Full-Light/Low	unknown	y	
AT3G14350	SRF7	Chr3:4786505	Full-Light/Low		y	protein phosphorylation, transmembrane receptor protein tyrosine kinase signaling pathway
AT3G47990	SIS3	Chr3:17718905	Full-Light/High		y	glucuronoxylan metabolic process, protein ubiquitination, response to high light intensity, response to hydrogen peroxide, sugar mediated signaling pathway, xylan biosynthetic process
AT3G48000	ALDH2	Chr3:17718905	Full-Light/High		y	metabolic process, oxidation-reduction process, response to cadmium ion

AT3G48010	CNGC16	Chr3:17718905	Full-Light/High		n	ion transport, transmembrane transport
**AT3G59020**		Chr3:21818882	Full-Light/High		y	intracellular protein transport, protein import into nucleus, docking
**AT3G59030**	TT12	Chr3:21818882	Full-Light/High		y	drug transmembrane transport, maintenance of seed dormancy, proanthocyanidin biosynthetic process, purine nucleobase transport, transmembrane transport
**AT3G59040**		Chr3:21818882	Full-Light/High	Tetratricopeptide repeat-like superfamily protein	y	chloroplast organization, pentose-phosphate shunt, rRNA processing, tRNA metabolic process
AT4G12240		Chr4:7287800	Full-Light/High		y	regulation of transcription, DNA-dependent
AT4G12250	GAE5	Chr4:7287800	Full-Light/High		y	carbohydrate metabolic process, cellular metabolic process, cellular response to phosphate starvation, cellular response to water deprivation, galactolipid biosynthetic process, nucleotide-sugar metabolic process
**AT4G13180**		Chr4:7657583	Full-Light/High		y	metabolic process, response to arsenic-containing substance
AT4G15450		Chr4:8841131, Chr4:8843014, Chr4:8843150	Full-Light/High, Full-Light/Low	Senescence/dehydration-associated protein-related	n	
AT4G15460		Chr4:8841131, Chr4:8843014, Chr4:8843150	Full-Light/High, Full-Light/Low	glycine-rich protein	n	
AT4G24790		Chr4:12776709	Full-Light/High		y	DNA replication
**AT5G28680**	ANX2	Chr5:10723903	Dark/Low		y	protein phosphorylation
**AT5G28690**		Chr5:10723903	Dark/Low	unknown	n	
AT5G41010	NRPB12	Chr5:16425024	Full-Light/Low		y	RNA splicing, via endonucleolytic cleavage and ligation, transcription from RNA polymerase II promoter, transcription, DNA-dependent

AT5G55340		Chr5:22442725	Full-Light/High	membrane bound O-acyl transferase family protein	y	
AT5G55350		Chr5:22442725	Full-Light/High	membrane bound O-acyl transferase family protein	n	
AT5G55360		Chr5:22442725	Full-Light/High	membrane bound O-acyl transferase family protein	n	
AT5G66690	UGT72E2	Chr5:26627873	Full-Light/High		y	Golgi vesicle transport, RNA methylation, cell wall modification, cellulose biosynthetic process, plant-type cell wall organization

[a]TAIR gene identifier

[b] y = gene is expressed in the seed or embryo, n = not known to be expressed in embryo or seed.
