# Supplementary material for: Association Mapping of Germination Traits in Arabidopsis thaliana Under Light and Nutrient Treatments: Searching for G×E Effects
Source: G3 (Bethesda). 2014 Jun 5;4(8):1465–78. doi: 10.1534/g3.114.012427 (PMC4132177; doi:10.1534/g3.114.012427)
Supplement: Supporting Information [file supp_g3.114.012427_TableS6.pdf]

Table S6 Enrichment (or lack thereof) for SNPs linked to candidate genes in the top 50, 100, etc. SNPs for TMAX. Enrichment was never significant based on 10000 permutations.

	Enrichment					
**Model**	**50**	**100**	**250**	**500**	**1000**	**5000**
Dark/Low	0	0	0	1.18	1.06	0.59
Dark/High	0	0	0.47	0.94	0.94	0.92
Full-Light/Low	0	0	0.47	0.47	0.35	0.87
Full-Light/High	2.39	2.39	1.90	1.42	1.78	1.01
Full	2.39	1.18	0.94	0.47	0.82	0.82
Genotype Only	2.39	1.18	0.47	0.47	0.71	0.80
GxE	0	0	0.47	0.71	1.06	0.78
GxL	0	1.18	0.94	0.94	1.06	1.06
GxN	0	0	0	0.47	0.35	0.61
