# Supplementary material for: Association Mapping of Germination Traits in Arabidopsis thaliana Under Light and Nutrient Treatments: Searching for G×E Effects
Source: G3 (Bethesda). 2014 Jun 5;4(8):1465–78. doi: 10.1534/g3.114.012427 (PMC4132177; doi:10.1534/g3.114.012427)
Supplement: Supporting Information [file supp_g3.114.012427_TableS7.pdf]

Table S7 Genes considered linked to significant reaction norm SNPs (see manuscript for details), the SNP(s) they are linked to, and model in which the significant SNP was found. Names, descriptions, expression, and GO information from TAIR.

Gene[a]	Name	SNP	Norm	Description	Expressed[b]	GO Biological Process
AT1G61890		Chr1:22870338	FPG, Nutrient under Full	MATE efflux family protein	y	drug transmembrane transport, jasmonic acid metabolic process, proline transport, response to abscisic acid stimulus, response to chitin, response to jasmonic acid stimulus, response to karrikin, response to salt stress, transmembrane transport
AT2G05755		Chr2:2177432	FPG, Light under Low	Nucleotide/sugar transporter family protein	y	sphingoid biosynthetic process, sterol biosynthetic process
AT2G24210	TPS10	Chr2:10297188	FPG, Nutrient under Full		y	meristem development, metabolic process, monoterpenoid biosynthetic process, response to jasmonic acid stimulus, response to wounding
AT2G24220	PUP5	Chr2:10297188	FPG, Nutrient under Full		y	nucleobase-containing compound transport
AT2G24230		Chr2:10297188	FPG, Nutrient under Full		y	protein phosphorylation, transmembrane receptor protein tyrosine kinase signaling pathway
AT4G18250		Chr4:10089582	TMAX, Light under High	receptor serine/threonine kinase	n	protein phosphorylation
AT5G07315	pre-tRNA	Chr5:2319344	FPG, Light under Low	pre-Tyr	n	translational elongation
AT5G07320	APC3	Chr5:2319344	FPG, Light under Low		y	ATP transport, Golgi localization, actin filament-based movement, mitochondrion localization, peroxisome localization, transmembrane transport, transport
AT5G07322		Chr5:2319344	FPG, Light	other RNA	n	unknown

			under Low			
AT5G07330		Chr5:2319344	FPG, Light under Low		n	unknown
AT5G07340		Chr5:2319344	FPG, Light under Low	Calreticulin family protein	y	protein folding, response to endoplasmic reticulum stress, response to heat, response to high light intensity, response to hydrogen peroxide
AT5G07350	TUDOR1	Chr5:2319344	FPG, Light under Low		y	Golgi vesicle transport, cellulose biosynthetic process, gene silencing by RNA, protein secretion, response to cadmium ion, response to salt stress
AT5G19095	pre-tRNA	Chr5:6399525	FPG, Nutrient under Full	pre-Gly	n	
AT5G39880		Chr5:15976193	FPG, Nutrient under Full	unknown	y	
AT5G39890		Chr5:15976193	FPG, Nutrient under Full		y	cell wall macromolecule metabolic process, oxidation-reduction process, regulation of hydrogen peroxide metabolic process, response to hypoxia, salicylic acid mediated signaling pathway
AT5G39895	pre-tRNA	Chr5:15976193	FPG, Nutrient under Full	pre-Ala	n	
AT5G39900		Chr5:15976193	FPG, Nutrient under Full	Small GTP-binding protein	y	
AT5G39910		Chr5:15976193	FPG, Nutrient under Full	Pectin lyase-like superfamily protein	n	carbohydrate metabolic process

[a]TAIR gene identifier

[b] y = gene is expressed in the seed or embryo, n = not known to be expressed in embryo or seed.
