# Supplementary material for: Association Mapping of Germination Traits in Arabidopsis thaliana Under Light and Nutrient Treatments: Searching for G×E Effects
Source: G3 (Bethesda). 2014 Jun 5;4(8):1465–78. doi: 10.1534/g3.114.012427 (PMC4132177; doi:10.1534/g3.114.012427)
Supplement: Supporting Information [file supp_g3.114.012427_FileS1.pdf]

Alboresi, A., C. Gestin, M.-T. Leydecker, M. Bedu, C. Meyer *et al.*, 2005 Nitrate, a signal relieving seed dormancy in *Arabidopsis*. Plant Cell and Environment **28:** 500–512.

Atwell, S., Y. S. Huang, B. J. Vilhjalmsson, G. Willems, M. Horton *et al.*, 2010 Genome-wide association study of 107 phenotypes in *Arabidopsis thaliana* inbred lines. Nature **465:** 627–631.

Aukerman, M., M. Hirschfeld, L. Wester, M. Weaver, T. Clack *et al.*, 1997 A deletion in the PHYD gene of the *Arabidopsis* Wassilewskija ecotype defines a role for phytochrome D in red/far-red light sensing. The Plant Cell **9:** 1317–1326.

Bentsink, L., J. Jowett, C. J. Hanhart, and M. Koornneef, 2006 Cloning of DOG1, a quantitative trait locus controlling seed dormancy in *Arabidopsis*. PNAS **103:** 17042–17047.

Cadman, C. S. C., P. E. Toorop, H. W. M. Hilhorst, and W. E. Finch-Savage, 2006 Gene expression profiles of *Arabidopsis* Cvi seeds during dormancy cycling indicate a common underlying dormancy control mechanism. The Plant Journal **46:** 805–822.

Chopin, F., M. Orsel, M.-F. Dorbe, F. Chardon, H.-N. Truong *et al.*, 2007 The *Arabidopsis* ATNRT2.7 nitrate transporter controls nitrate content in seeds. The Plant Cell **19:** 1590–1602.

Debeaujon, I., K. M. Léon-Kloosterziel, and M. Koornneef, 2000 Influence of the testa on seed dormancy, germination, and longevity in *Arabidopsis*. Plant Physiology **122:** 403–414.

Dill, A., and T. Sun, 2001 Synergistic derepression of gibberellin signaling by removing RGA and GAI function in *Arabidopsis thaliana*. Genetics **159:** 777–785.

Duque, P., and N. Chua, 2003 IMB1, a bromodomain protein induced during seed imbibition, regulates ABA- and phyA-mediated responses of germination in *Arabidopsis.* The Plant Journal **35:** 787–799

Finch-Savage, W. E., C. S. C. Cadman, P. E. Toorop, J. R. Lynn, and H. W. M. Hilhorst, 2007 Seed dormancy release in *Arabidopsis* Cvi by dry after-ripening, low temperature, nitrate and light shows common quantitative patterns of gene expression directed by environmentally specific sensing. The Plant Journal **51:** 60–78.

Finkelstein, R. R., 1994 Maternal effects govern variable dominance of two abscisic acid response mutations in *Arabidopsis thaliana*. Plant Physiology **105:** 1203–1208.

Griffiths, J., K. Murase, I. Rieu, R. Zentella, Z.-L. Zhang *et al.*, 2006 Genetic characterization and functional analysis of the GID1 gibberellin receptors in *Arabidopsis*. The Plant Cell **18:** 3399–3414.

Hennig, L., W. Stoddart, M. Dieterle, G. Whitelam, and E. Schafer, 2002 Phytochrome E controls light-induced germination of *Arabidopsis*. Plant Physiology **128:** 194–200.

Holman, T. J., P. D. Jones, L. Russell, A. Medhurst, S. U. Tomas *et al.*, 2009 The N-end rule pathway promotes seed germination and establishment through removal of ABA sensitivity in *Arabidopsis*. PNAS **106:** 4549–4554.

Jacobsen, S. E., and N. E. Olszewski, 1993 Mutations at the SPINDLY locus of *Arabidopsis* alter gibberellin signal-transduction. The Plant Cell **5:** 887–896.

Kim, D. H., S. Yamaguchi, S. Lim, E. Oh, J. Park *et al.*, 2008 SOMNUS, a CCCH-type zinc finger protein in *Arabidopsis*, negatively regulates light-dependent seed germination downstream of PIL5. The Plant Cell **20:** 1260–1277.

Koornneef, M., M. L. Jorna, D. Derswan, and C. Karssen, 1982 The isolation of abscisic-acid (ABA) deficient mutants by selection of induced revertants in non-germinating gibberellin sensitive lines of *Arabidopsis thaliana* (L.) Heynh. Theoretical and Applied Genetics **61:** 385–393.

Koornneef, M., C. J. Hanhart, H. W. M. Hilhorst, and C. Karssen, 1989 *In vivo* inhibition of seed development and reserve protein accumulation in recombinants of abscisic acid biosynthesis and responsiveness mutants in *Arabidopsis thaliana*. Plant Physiology **90:** 463–469.

Kucera, B., M. Cohn, and G. Leubner-Metzger, 2005 Plant hormone interactions during seed dormancy release and germination. Seed Science Research **15:** 281–307.

Lee, S., H. Cheng, K. King, W. Wang, Y. He *et al.*, 2002 Gibberellin regulates *Arabidopsis* seed germination via RGL2, a GAI/RGA-like gene whose expression is up-regulated following imbibition. Genes & Development **16:** 646–658.

Liu, Y., M. Koornneef, and W. J. J. Soppe, 2007 The absence of histone H2B monoubiquitination in the *Arabidopsis* hub1 (rdo4) mutant reveals a role for chromatin remodeling in seed dormancy. The Plant Cell **19:** 433–444.

Martinez-Garcia, J., E. Huq, and P. Quail, 2000 Direct targeting of light signals to a promoter element-bound transcription factor. Science **288:** 859–863.

Oh, E., J. Kim, E. Park, J. Kim, C. Kang *et al.*, 2004 PIL5, a phytochrome-interacting basic helix-loop-helix protein, is a key negative regulator of seed germination in *Arabidopsis thaliana*. The Plant Cell **16:** 3045–3058.

Penfield, S., E. Josse, R. Kannangara, A. Gilday, K. Halliday *et al.*, 2005 Cold and light control seed germination through the bHLH transcription factor SPATULA. Current Biology **15:** 1998–2006.

Raz, V., J. Bergervoet, and M. Koornneef, 2001 Sequential steps for developmental arrest in *Arabidopsis* seeds. Development **128:** 243–252.

Ren, Z., Z. Zheng, V. Chinnusamy, J. Zhu, X. Cui *et al.*, 2010 RAS1, a quantitative trait locus for salt tolerance and ABA sensitivity in *Arabidopsis*. PNAS **107:** 5669–5674.

Russell, L., V. Larner, S. Kurup, S. Bougourd, and M. Holdsworth, 2000 The *Arabidopsis* COMATOSE locus regulates germination potential. Development **127:** 3759–3767.

Shinomura, T., A. Nagatani, J. Chory, and M. Furuya, 1994 The induction of seed germination in *Arabidopsis thaliana* is regulated principally by Phytochrome B and secondarily by Phytochrome A. Plant Physiology **104:** 363–371.

Steber, C. M., S. E. Cooney, and P. McCourt, 1998 Isolation of the GA-response mutant sly1 as a suppressor of ABI1-1 in *Arabidopsis thaliana*. Genetics **149:** 509–521.

Zentella, R., Z.-L. Zhang, M. Park, S. G. Thomas, A. Endo *et al.*, 2007 Global analysis of DELLA direct targets in early gibberellin signaling in *Arabidopsis*. The Plant Cell **19:** 3037–3057.
